# Low-cost, local production of a safe and effective disinfectant for resource-constrained communities

**DOI:** 10.1371/journal.pgph.0002213

**Published:** 2024-06-25

**Authors:** Andrea Naranjo-Soledad, Logan Smesrud, Siva R. S. Bandaru, Dana Hernandez, Meire Mehare, Sara Mahmoud, Vijay Matange, Bakul Rao, Chandana N., Paige Balcom, David Olugbenga Omole, César Álvarez-Mejía, Varinia López-Ramrez, Ashok Gadgil

**Affiliations:** 1 Department of Civil and Environmental Engineering, University of California, Berkeley, Berkeley, California, United States of America; 2 The Molecular Foundry, Lawrence Berkeley National Laboratory, Berkeley, California, United States of America; 3 Department of Chemical and Nuclear Engineering, University of California, Berkeley, Berkeley, California, United States of America; 4 VINYAS Architects, Urban Designers, Landscape Architects, Delhi, India; 5 Centre for Technology Alternatives for Rural Areas, Indian Institute of Technology Bombay, Mumbai, Maharashtra, India; 6 Centre for Emerging Technologies for Sustainable Development, Indian Institute of Technology Jodhpur, Jodhpur, Rajasthan, India; 7 Engineering R&D, Takataka Plastics, Gulu, Uganda; 8 Biosystems Engineering, Gulu University, Gulu, Uganda; 9 Department of Civil Engineering, Covenant University, Ota, Ogun State, Nigeria; 10 School of Civil and Environmental Engineering, University of the Witwatersrand, Johannesburg, South Africa; 11 Department of Environmental Engineering, Tecnológico Nacional de México, ITS de Abasolo, Abasolo, Guanajuato, Mexico; 12 Department of Biochemical Engineering, Tecnológico Nacional de México/ITS de Irapuato, Irapuato, Guanajuato, Mexico; Indian Institute of Public Health Shillong, INDIA

## Abstract

Improved hygiene depends on the accessibility and availability of effective disinfectant solutions. These disinfectant solutions are unavailable to many communities worldwide due to resource limitations, among other constraints. Safe and effective chlorine-based disinfectants can be produced via simple electrolysis of salt water, providing a low-cost and reliable option for on-site, local production of disinfectant solutions to improve sanitation and hygiene. This study reports on a system (herein called “Electro-Clean”) that can produce concentrated solutions of hypochlorous acid (HOCl) using readily available, low-cost materials. With just table salt, water, graphite welding rods, and a DC power supply, the Electro-Clean system can safely produce HOCl solutions (~1.5 liters) of up to 0.1% free chlorine (i.e.,1000 ppm) in less than two hours at low potential (5 V DC) and modest current (~5 A). Rigorous testing of free chlorine production and durability of the Electro-Clean system components, described here, has been verified to work in multiple locations around the world, including microbiological tests conducted in India and Mexico to confirm the biocidal efficacy of the Electro-Clean solution as a surface disinfectant. Cost estimates are provided for making HOCl locally with this method in the USA, India, and Mexico. Findings indicate that Electro-Clean is an affordable alternative to off-the-shelf commercial chlorinator systems in terms of first costs (or capital costs), and cost-competitive relative to the unit cost of the disinfectant produced. By minimizing dependence on supply chains and allowing for local production, the Electro-Clean system has the potential to improve public health by addressing the need for disinfectant solutions in resource-constrained communities.

## Introduction

**Fig 1:**
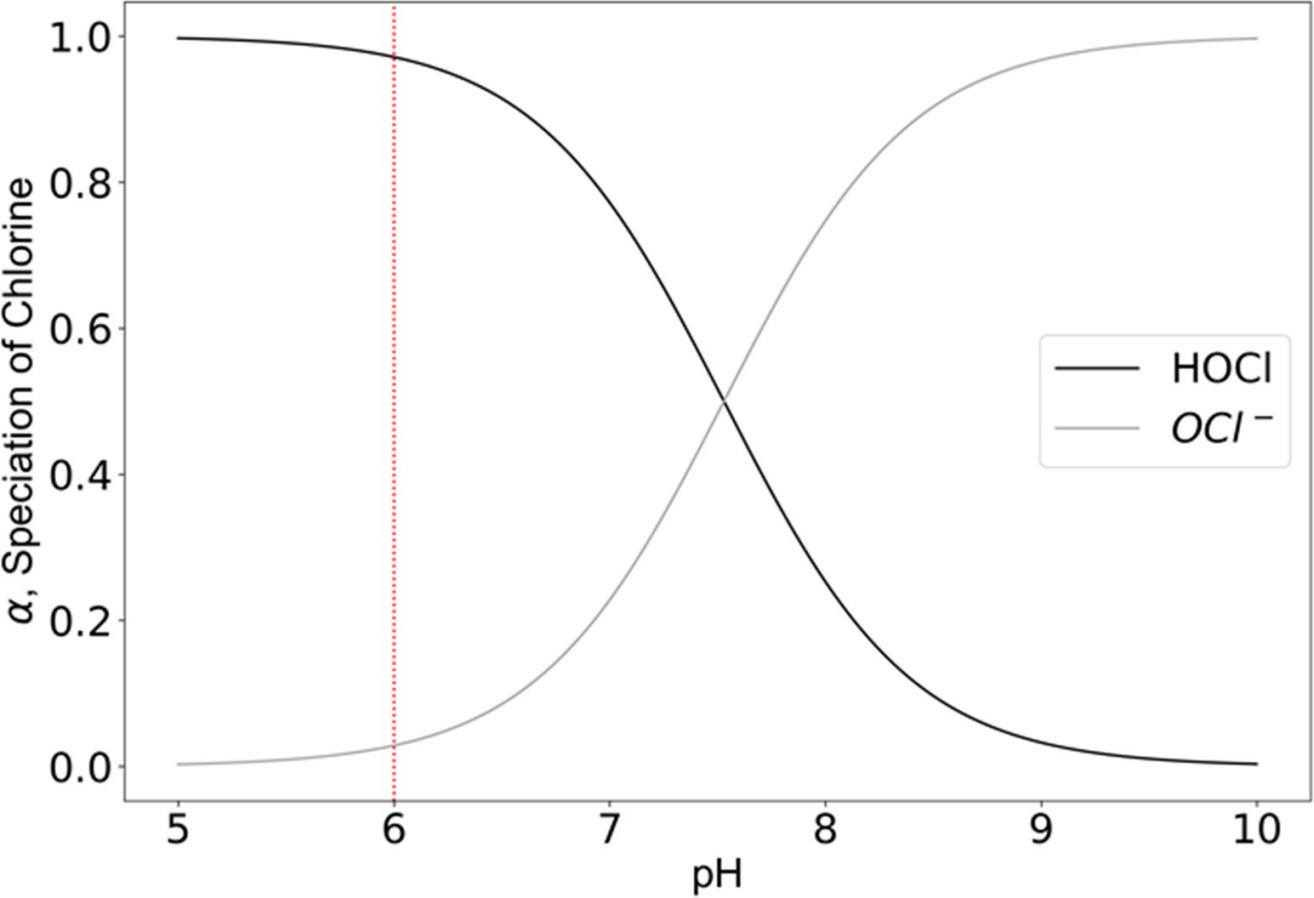
Speciation of aqueous chlorine species (represented by the fractional alpha value) as a function of pH, around circumneutral pH values. In the pH range shown above, the predominant species is hypochlorous acid at the lower end and shifts to the hypochlorite ion at the upper end. The vertical line shown at pH 6 is the target pH value for a disinfectant solution where hypochlorous acid is the predominant species. This figure was produced using published chlorine speciation equations [17]. Alpha represents the fractional concentration of a chlorine species over the total chlorine concentration.

Chlorine-based disinfectants are widely used across a variety of settings and applications, including hospitals, public spaces, food preparation, and drinking water disinfection [1–4]. Even when disinfectants are available, due to cost and limited supply chains, these are often diluted excessively as a way to ration the solution, making it less effective [5, 6]. The efficacy of chlorine-based disinfectants is also highly pH-dependent. At a pH below 4, chlorine exists primarily as dissolved chlorine gas. As shown in Fig 1, at a pH of 6, chlorine exists in water mostly as hypochlorous acid (HOCl), and as pH increases above 8, the dominant species shifts to hypochlorite (OCl-). These different species have varying disinfecting powers, with HOCl being 80 to 100 times more effective at bacterial inactivation than the hypochlorite ion (OCl-) [7].

Numerous studies have looked at the virucidal effect of HOCl solutions, demonstrating that a concentration as low as 20 ppm has virucidal power [8, 9]. Furthermore, unlike bleach solutions (i.e. 3–8 wt% NaOCl), hypochlorous acid is not a skin irritant and therefore safer to handle [10–13]. The amplification of biocidal properties in HOCl compared to NaOCl illustrates the importance of pH adjustment for chlorine-based disinfectants [14–16].

Chlorine gas hydrolyzes to hypochlorous acid quite rapidly, and proceeds as follows:

$${Cl}_{2}(g)+H_{2}O\to HOCl+HCl$$ (Eq 1)

Hypochlorous acid can proceed to dissociate to the hypochlorite ion, as follows:

$$HOCl\to H^{+}+{OCl}^{-}$$ (Eq 2)

Hypochlorous acid solutions can be produced by 1) bubbling chlorine gas into water, 2) acidifying sodium hypochlorite (bleach), and 3) electrolysis of saline water [14, 18, 19]. The approach of bubbling chlorine gas into water requires access to compressed gas cylinders, the handling of which requires skilled training and still presents significant safety concerns [20, 21]. The acidification of bleach is simple, yet requires access to and careful handling of bleach, which is commercially available at concentrations that cause eye and skin irritation [21]. Furthermore, it requires careful pH adjustment as lowering the pH below 4 would result in the release of chlorine gas, a harmful substance that can cause serious irritation when inhaled [20].

On-site production of hypochlorous acid via electrolysis of salt water is a safer, commonly-used alternative that only uses ordinary table salt (sodium chloride) and electricity as inputs, thus avoiding supply chain issues [22–24]. Furthermore, numerous products using this technique—known as electrochlorinators, are commercially available [25–27]. However, most commercially available electrochlorinators use expensive electrode materials, such as dimensionally stable mixed metal oxide (MMO) electrocatalyst anodes consisting of iridium and ruthenium oxides such as RuO_2_^-^ and IrO_2_^-^ [28–30]. While MMO anodes have high efficiency in electrochemical chlorine generation, their high costs make electrochlorinators inaccessible to resource-limited communities.

Prior efforts to produce chlorine-based disinfectant solutions using low-cost materials include a student team from the Massachusetts Institute of Technology (MIT) that focused on the use of carbon rods obtained from zinc-carbon batteries, a simple saline solution, and a recycled plastic cup as the container [31]. Although this design was quite inexpensive and effective at producing free chlorine, it required the extraction of carbon rods from zinc-carbon batteries. From experiences with collaborators in various countries in non-academic settings, significant resistance and concern was observed regarding the process of breaking open a zinc-carbon battery to extract the carbon rod, despite reassurances of its harmlessness. This user feedback highlighted the importance of minimal complexity, one of the key characteristics of technology adoption rate as defined by Rogers and other researchers on the Diffusion of Innovations [32]. Further improvements to the design developed by the team at MIT were discontinued since its complexity would likely lower the rate of technology adoption. As a result, the design efforts shifted to focus on minimizing complexity to the end-user, even if that slightly increased the cost of production.

This paper reports on a simple electrochemical process (herein called “Electro-Clean” for short) that aims to eliminate these economic and accessibility barriers, thereby improving access to best practices of hygiene in resource-poor environments. The development of Electro-Clean was initiated in response to COVID-19, as this global pandemic highlighted the importance of local disinfectant supply options. Electro-Clean has low capital and operating costs, and produces a powerful, safe-to-use hypochlorous acid disinfectant solution, with hypochlorous acid being an approved disinfectant by the World Health Organization (WHO), U.S. Environmental Protection Agency (EPA), and Centers for Disease Control and Prevention (CDC) [33–35]. The Electro-Clean process can produce HOCl solutions at concentrations sufficient for addressing surface disinfection, producing 1.5 liters solution with up to 0.1% hypochlorous acid (i.e. 1000 ppm) in less than two hours, operating at low potential (5V DC) and modest current (around 5A). The development and testing of Electro-Clean involved collaboration with co-authors and other project partners in seven countries (US, Mexico, India, Nigeria, Uganda, Mali, and Mozambique) spanning three continents, demonstrating how this process can be replicated in a variety of contexts with affordable, locally available materials.

The objective of this paper is to evaluate the performance, cost-effectiveness, and reproducibility of the Electro-Clean process, aiming to present it as a viable alternative for on-site generation of a safe disinfectant in resource-limited settings.

## Materials and methods

### Reactor materials and designs

#### Electrolyte

The electrolyte solutions were prepared using either reagent-grade NaCl, or commercial-grade NaCl, and local municipal tap water. In Berkeley, California, tap water is supplied by the East Bay Municipal Utility District, and its composition is cited in their water quality report [36]. Note that the water used to prepare the salt water solution should always be free of organic content as this can rapidly react with hypochlorous acid and lower its disinfecting power [37, 38].

To ease the reproducibility of the results at UC Berkeley by others, non-iodized salt was used in all experiments reported here, unless stated otherwise. An electrolyte solution of 30,000 mg/L NaCl (a similar salinity to that of seawater) was used for all experiments, unless stated otherwise.

#### Power supply

Power supplies (Switched-Mode Power Supplies, SMPS) used in computers have become cheaper and more efficient with the large-scale manufacturing of these devices. They perform high-efficiency conversion of grid power into a 5-volt supply with high current capacity (e.g. 20 A or 40 A). Therefore, an SMPS was used to supply the low potential required to drive the electrolysis reaction. The SMPS (MEISHILE brand, Amazon.com, country of origin: China) supplied 5 volts DC, with a maximum output current of 40 amperes.

#### Electrodes

**Fig 2:**
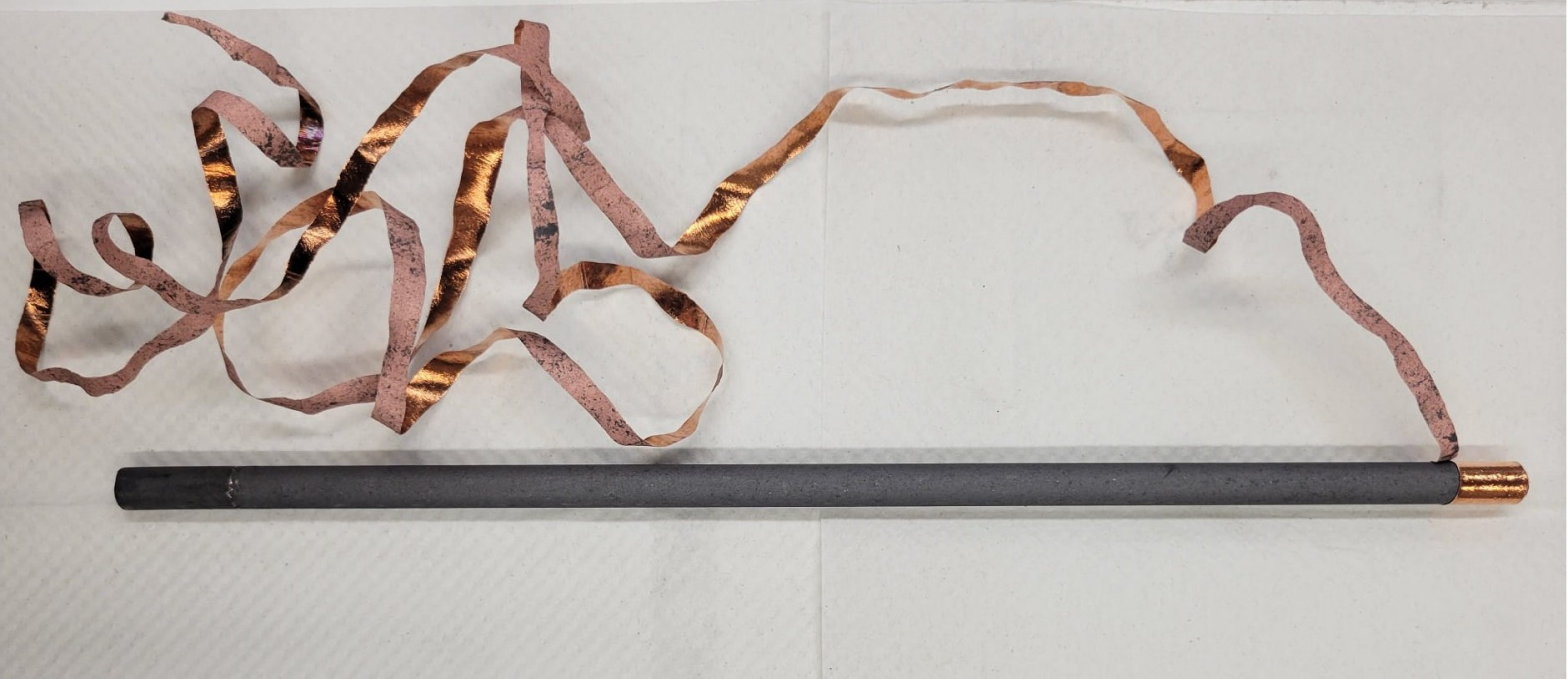
Copper peeled off as a winding strip from the copper-coated carbon gouging rod.

Uncoated bare carbon gouging rods, 9.52 mm (3/8 in) in diameter and 30.5 cm (12 in) in length (McMaster-Carr) served as both the anode and cathode. Before use, the electrodes were rinsed with tap water and brushed with a nylon brush to remove loose carbon particles. The carbon gouging rods, shown in Fig 2, allow for chlorine production while gradually degrading due to oxidation [39–41]. Despite the undesirability of electrode disintegration, the use of low-cost carbon is preferred over other alternatives such as stainless steel, which rusts in the presence of chloride ions [42–44]. Expensive and locally unavailable high-performance electrodes, such as Iridium-Ruthenium Mixed Metal Oxides (MMO), are not considered viable for the Electro-Clean system.

In Mexico and Nigeria, co-authors were only able to find carbon gouging rods with copper coating. Therefore, chlorine production using copper-coated carbon gouging rods was also investigated in this study. The copper coating was peeled off with small pliers (Fig 2), leaving copper coating only at the tip of the rod where the electrical connections are made.

#### Filtration of carbon slurry

Disintegration of the anodic carbon rod occurs during electrolysis, releasing carbon particles in the electrolyte solution [39–41]. Although the amount of free chlorine produced is not affected by the presence of these particles, the cloudy physical appearance is undesirable for a cleaning product. Nylon cloth and coffee filters were tested to remove these suspended carbon particles from the final hypochlorous acid solution. For the nylon cloth, the filter material was secured around the anode during electrolysis to prevent the carbon particles from escaping into the bulk solution. For the coffee filter, the solution at the end of electrolysis was poured over the filter for particle removal. The concentration of free chlorine was measured with and without the use of filters for comparison.

#### Reactor assembly and operation

**Fig 3:**
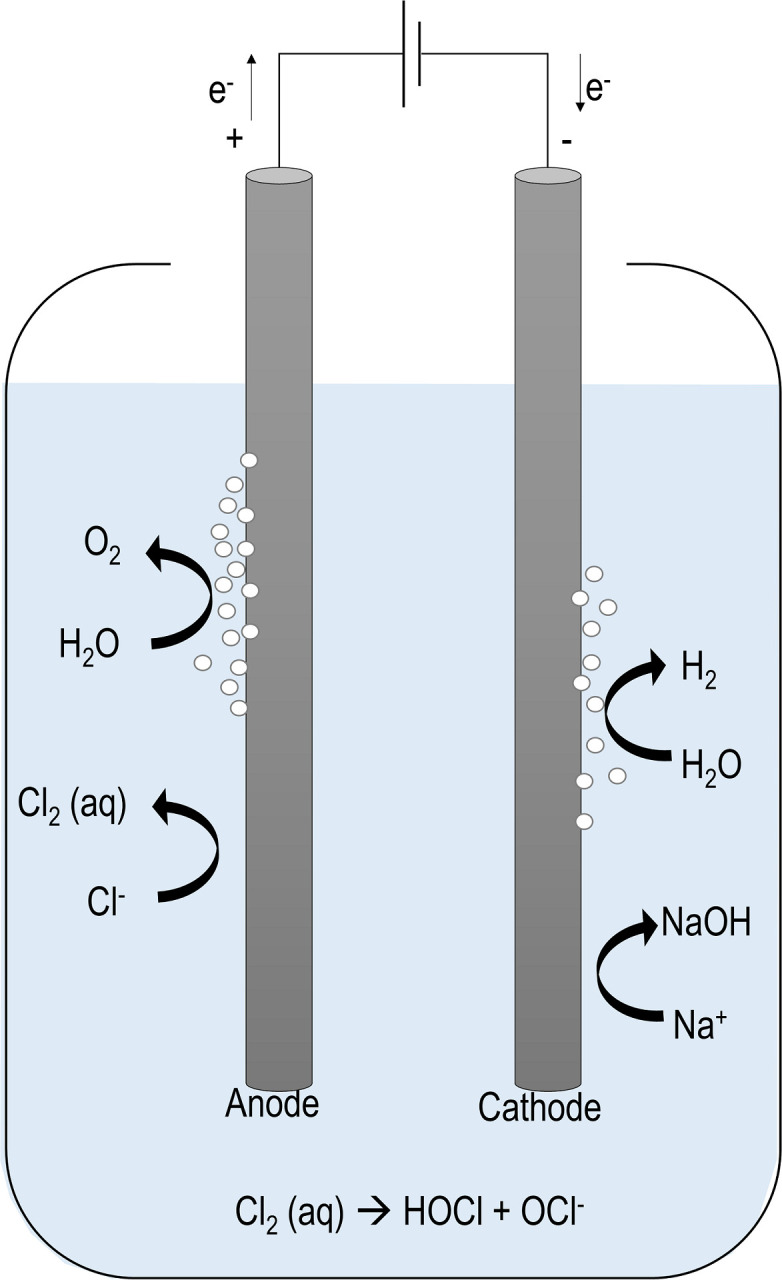
Schematic of Electro-Clean reactor system. The Electro-Clean reactor system consists of two carbon gouging rods as electrodes, 30,000 mg/L sodium chloride dissolved in tap water as the electrolyte solution, and an externally applied potential that drives the reaction to produce chlorine gas on the anode, which gets immediately hydrolyzed to produce hypochlorous acid and/or hypochlorite in the electrolyte.

**Fig 4:**
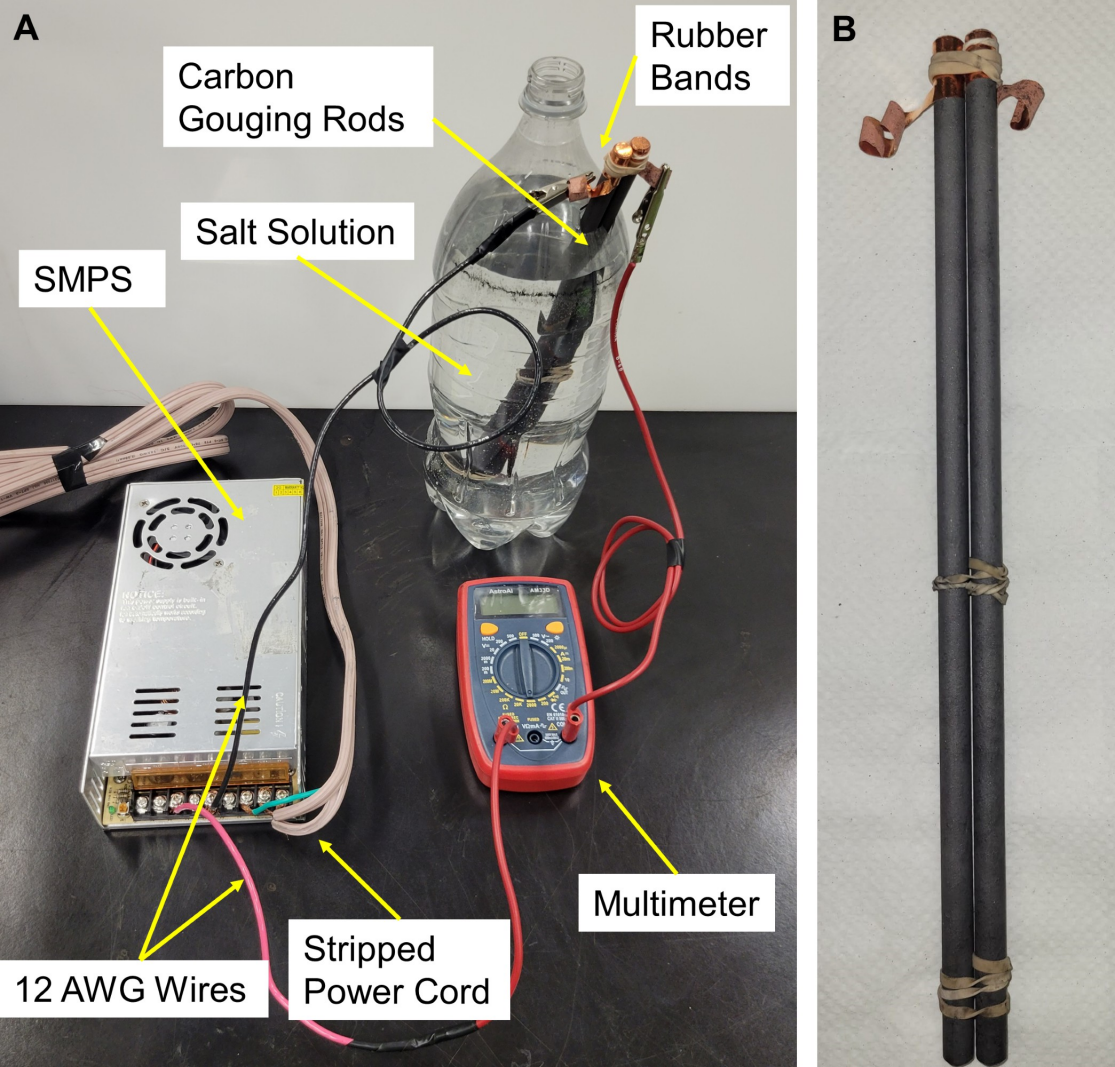
Digital picture of the Electro-Clean assembly. (A) Electro-Clean batch reactor assembly, and (B) Carbon gouging electrode assembly.

The simplest reactor assembly for the Electro-Clean process consisted of a 1.5-liter soda bottle, carbon gouging electrodes, a multimeter, a SMPS, and a 30,000 mg/L table salt electrolyte solution prepared with tap water. A 12-gauge power cord with a three-prong plug on one side was used to connect the SMPS to the power outlet. The multimeter was connected in series to measure current, and a 12 AWG insulated electrical wire was used for making connections between the electrodes and the SMPS. The Electro-Clean reactor assembly schematic and digital pictures are shown in Figs 3 and 4A. The two carbon gouging electrodes were aligned in parallel and held tightly together with rubber bands, which also served as spacers between the electrodes to prevent short-circuiting, as shown in Fig 4B.

As seen in Fig 4A, the bottle’s upper sidewall was cut to make an opening to insert the carbon electrode rods. The reactor was operated in batch for 90 minutes with an externally supplied direct current of 5 volts. This resulted in a supplied current of about 5 amps, depending on the internal resistance of the circuit connections.

#### Other size scales considered for Electro-Clean

In addition to the 1.5-liter soda bottle design, four other designs were explored: 1) a smaller, household-scale design producing 250 mL of concentrated HOCl solution, 2) a larger, community-scale design capable of producing 15 L of dilute HOCl solution, 3) a co-axial electrodes assembly, and 4) a reactor using a 1.5-liter soda bottle, but with multiple, parallel electrode assemblies, capable of producing the concentrated HOCl in a shorter time. For both the 250 mL and 15 L designs, stainless steel, and carbon rods were studied as the cathode and anode, respectively. A more detailed description of these alternative designs is in S3 Appendix.

### Quantitative measurements

#### Chlorine concentration measurements

An ultraviolet/visible light (UV-Vis) spectrophotometer (Hach DR6000) was used to measure the free chlorine concentration in the generated disinfectant through colorimetry at a wavelength of 530 nm. The N,N-diethyl-p-phenylenediamine, or DPD, reagent sachet (Hach brand) was added to 10 mL of the diluted disinfectant solution, producing a pink color when free chlorine is present. Due to the limited and low range of chlorine concentrations that can be measured using the DPD method, dilution factors ranged from 250x to 1000x depending on the experiment.

#### pH adjustment

Various safe and easily accessible acids were tested for their suitability to adjust the pH of the electrolyte and/or disinfectant solution, including distilled white vinegar (Heinz, 5% acidity, country of origin: USA), lemon juice, and lime juice. Early experiments showed that although the natural acidity of the lemon and lime juice could decrease the pH of the disinfectant solution to the desired value of 6, they were unsuitable due to their high content of organic matter which leads to the disappearance of free chlorine [37, 38].

For all experiments presented herein, unless stated otherwise, distilled white vinegar (i.e. dilute acetic acid) was used to adjust the pH. Hydrochloric acid (HCl, also known as muriatic acid), is an equally effective alternative for lowering pH, but it is less accessible in local markets due to safety concerns. Muriatic acid may have yielded slightly different observations and conclusions due to the possible interaction of acetic acid’s carboxyl functional group with electrolysis products. Nevertheless, these limitations were accepted given that vinegar is more widely available and therefore more suitable for the intended Electro-Clean users.

#### Faradaic efficiency tests

The Faradaic efficiency of an electrode is calculated as the ratio between the observed free-chlorine production (measured with the UV-Vis spectrophotometer) and the theoretical free chlorine production (calculated using Faraday’s Law). This parameter provides unique insight into the efficiency of chlorine production by the Electro-Clean process. A thorough explanation of the Faradaic efficiency calculations is presented in S1 Appendix.

### Durability of electrodes over time

During the electrolysis of a brine solution, carbon anode electrodes undergo oxidation, slow mechanical disintegration, and eventual failure [39, 40, 45]. This process occurs not only on the surface of the electrodes but also within their high-porosity structure. To slow the disintegration of the carbon rods during electrolysis, a technique of filling the pores with light mineral oil (commercial name: Johnson & Johnson Baby Oil) was explored. Each carbon gouging electrode was submerged in light mineral oil for 20 hours before use. Only the tips of the electrodes, where the connections to the power supply were made, were left unsubmerged.

The two performance parameters used to evaluate the durability of the electrode rod were 1) free chlorine concentration, and 2) current. The 1.5-liter reactor system design was used to observe the durability of the bare carbon rods as well as mineral-oil-soaked carbon rods. The electrolyte was a 1.35-liter solution of 30,000 mg/L NaCl. A peristaltic pump was used to continuously feed the reactor with electrolyte, and excess electrolyte was continuously removed from the reactor to maintain a constant volume. The flow rate of 0.25 mL/s led to a hydraulic residence time of 90 minutes in the reactor volume. All samples were taken at the outlet. Long-term experiments were carried out in triplicates for the bare carbon electrodes and the mineral-oil-soaked electrodes. See Fig A in S4 Appendix for more detail on the continuous-flow assembly.

To serve as a control for these long-term experiments, chlorine production by carbon electrodes in a single batch process was evaluated. These triplicate batch tests ran for 90-minute electrolysis times in 1.35-L batch volumes.

### Effect of electrolyte pH on free chlorine production

The impact of electrolyte pH pre-electrolysis on free chlorine production was examined by comparing an electrolyte solution with a semi-neutral pH of 8 to one with a slightly acidic pH of 5. The experiments were conducted in triplicates for each electrolyte pH condition. For each set of electrolysis condition experiments, 4.5-L of electrolyte stock solution of 30,000 mg/L as NaCl was prepared in a single container and then split evenly into three plastic water bottles (each 1.5-L capacity, with 1.35-L electrolyte solution). Distilled white vinegar was used to adjust the pH for the slightly acidic electrolyte. Following electrolysis for each set of experiments, the post-electrolysis pH was measured and adjusted to 6 using distilled white vinegar to ensure that all available chlorine was present as HOCl. The 1.35-liter Electro-Clean process assembly was used for this study, in batch mode, with an electrolysis time of 90 minutes.

### Free chlorine decay over time

Prior literature has noted that HOCl solutions are best stored in a dark, cool place, and in glass or plastic (not metal) containers to slow down the loss of free chlorine over time due to reconversion to a salt solution [46–48]. The decay of free chlorine in the produced disinfectant solution was studied when stored at room temperature in a dark closet. The material of the storage container (glass versus plastic) and the presence of vinegar (used for pH adjustment) were also considered.

A batch of the disinfectant solution was produced following the Electro-Clean process in a 2-liter plastic soda bottle using tap water (local to Berkeley). The free chlorine concentration of the produced disinfectant was 915 ± 35 ppm as Cl_2_. This was then diluted in half (~440 ppm), which is the concentration at which the chlorine decay experiments began. The diluted HOCl solution was split into four containers: two 16 fl-oz (473 mL) glass bottles, and two 16.9-fl-oz (500 mL) plastic bottles. Each container was filled near full capacity at the start of the experiment. About 3 mL of vinegar was added to lower the pH from 8.8 to 6.4 ± 0.1 in the plastic bottle and glass bottle. All four bottles were closed to prevent evaporative loss and periodically monitored for free chlorine over a month duration (34 days).

### Reproducibility in other communities worldwide

To ensure that the Electro-Clean process performed similarly in other contexts worldwide, co-authors in India, Mexico, Nigeria, and Uganda replicated the Electro-Clean process using reasonable substitutions of locally available materials. While the main components of the Electro-Clean process remained the same (e.g., carbon-gouging electrodes, 5-volt/40-amp SMPS, tap water, salt, vinegar, and a plastic container), the manufacturer of each component varied from community to community. In the case of Nigeria, the co-authors used dilute HCl for pH adjustment instead of white vinegar. S2 Table summarizes the manufacturer specifications of the materials used by co-authors outside the USA.

### Microbiological assays to evaluate the biocidal effect of the produced hypochlorous acid

To validate the disinfecting capabilities of the Electro-Clean solution, microbiological assays were conducted for its application on various high-touch material surfaces in public spaces and laboratory settings in Mexico, and laboratory settings in India. In each case, a hypochlorous acid solution was prepared by the Electro-Clean process. It was then diluted to the concentration of intended use (in this case, approximately 250 ppm for common surface disinfection), and subsequently adjusted with distilled white vinegar to pH 6 [8, 9, 49]. The materials and methods used for the microbiological studies performed in Mexico and India can be found in S5 and S6 Appendices, accordingly.

### Comparative cost analysis

**Fig 5:**
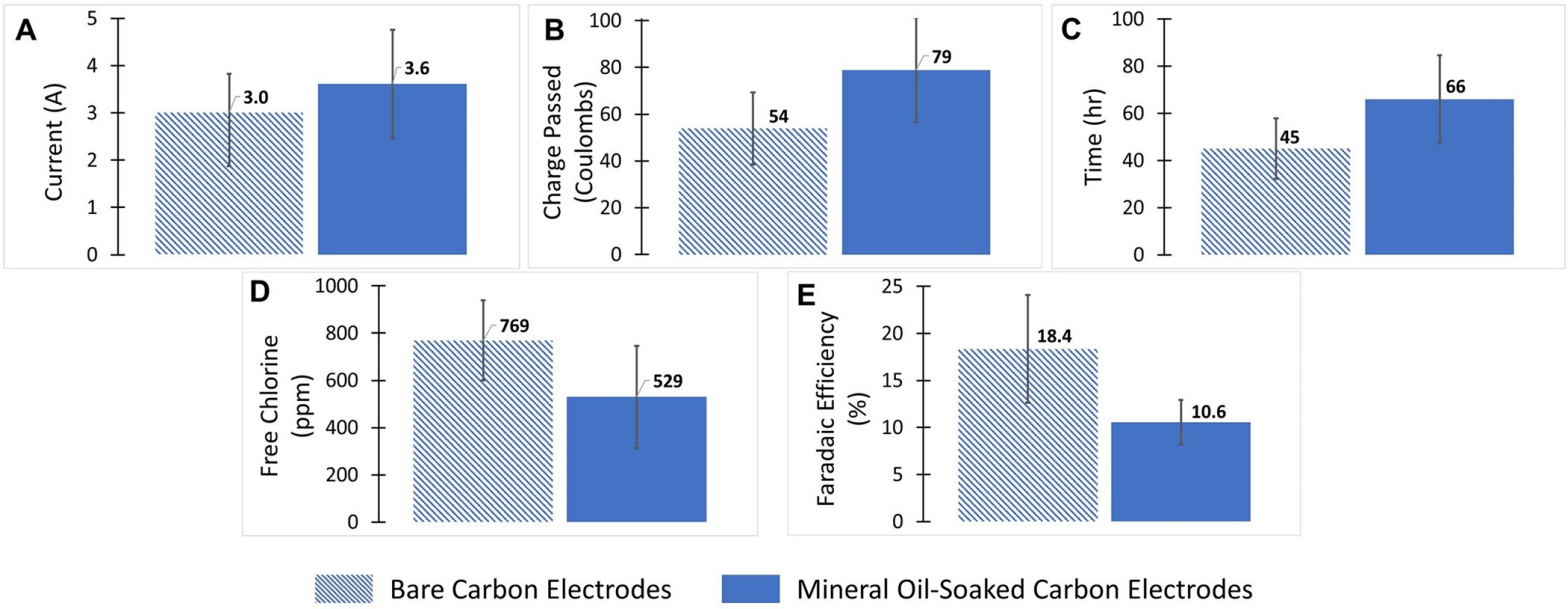
Long-term (~50 hours) performance of 1.5-liter Electro-Clean set-up using bare carbon electrodes and mineral-oil-soaked carbon electrodes. (A) Average current during long-term operation, (B) Average charge passed in Coulombs on the electrodes before failure, (C) Average time in hours of the anode lifespan, (D) Average free chlorine concentration produced for a residence time of 90 minutes in a 1.35-liter volume, (E) Average Faradaic efficiency for producing free chlorine throughout the long-term operation.

To quantify the cost of the Electro-Clean disinfectant production system, and to understand which items contribute to that total cost, an analysis evaluated both the capital and operating costs of Electro-Clean. To conduct this economic assessment and comparative analysis, spot prices as of April 2023 were obtained from three countries (USA, India, Mexico) for all reactor components. The Electro-Clean system was also compared against commercially available electrochlorinators and locally available chlorine-based disinfectant products. For this analysis, the expected lifespan of the electrodes reflects 90 hours of reactor operation, where sixty 1.35-L batches of 800 ppm free chlorine solution can be produced at an average current of 3.5 A and 5 V before complete exhaustion of the electrodes. These lifespan parameters come from the results shown in Fig 5, where bare carbon rods experienced an average 45-hour lifetime before failure while also accounting for the doubling of electrode lifetime by employing polarity reversal.

## Results and discussion

### Chlorine production by carbon electrodes in batch process

In a single anode/cathode configuration, repeated and averaged over triplicates, the following values were observed: 3.82 ± 0.14 ampere average current, 17.1 ± 0.3 percent Faradaic efficiency for free chlorine production, and 962 ± 20 ppm of total free chlorine production over 90-minute electrolysis times in 1.35-L batch volumes.

### Durability of electrodes over time

During the long-term experiments, Faradaic efficiency remained relatively constant, even right before the anode ruptured. Current, on the other hand, gradually decreased over time as the anode degraded. A reduction in current and visual thinning of the anode (due to disintegration) were indicators that the anode was soon to rupture.

The data as a function of charge passed (Coulombs) for the bare and mineral-oil-soaked electrodes from all three trials can be found in S4 Appendix. The average values for the three trials for bare and mineral-oil-soaked carbon electrodes, with error bars showing the standard deviation, are shown in Fig 5. Although the mineral-oil-soaked electrodes seemed to operate at a slightly higher average current as seen in Fig 5A, the difference in current is within the margin of error. Soaking the carbon rods in mineral oil allowed for a higher amount of charge to be sustained, resulting in a longer useful lifetime for the carbon electrode. Fig 5C demonstrates that the mineral-oil-soaked carbon rods can last approximately 20 hours (about 45%) longer than the bare carbon rods when operated at the same current. However, soaking the rods in mineral oil lowered the free chlorine concentration by 31%, as seen in Fig 5D. Correspondingly, soaking the rods in the mineral oil also lowered the Faradaic efficiency. This could be due to the mineral oil promoting other side reactions, such as oxygen evolution, at the anode.

Findings from the long-term experiments described here and presented in Fig 5 suggest that although the electrode life can be prolonged by submerging the anode in mineral oil, the advantage is offset by a reduction in free chlorine production. As a result, this approach is not recommended. However, future studies could investigate other low-cost, readily available materials to enhance the durability of carbon gouging electrodes, as it is an important aspect of improving the sustainability and cost-effectiveness of this disinfectant production process.

### Electrode disintegration during electrolysis

**Fig 6:**
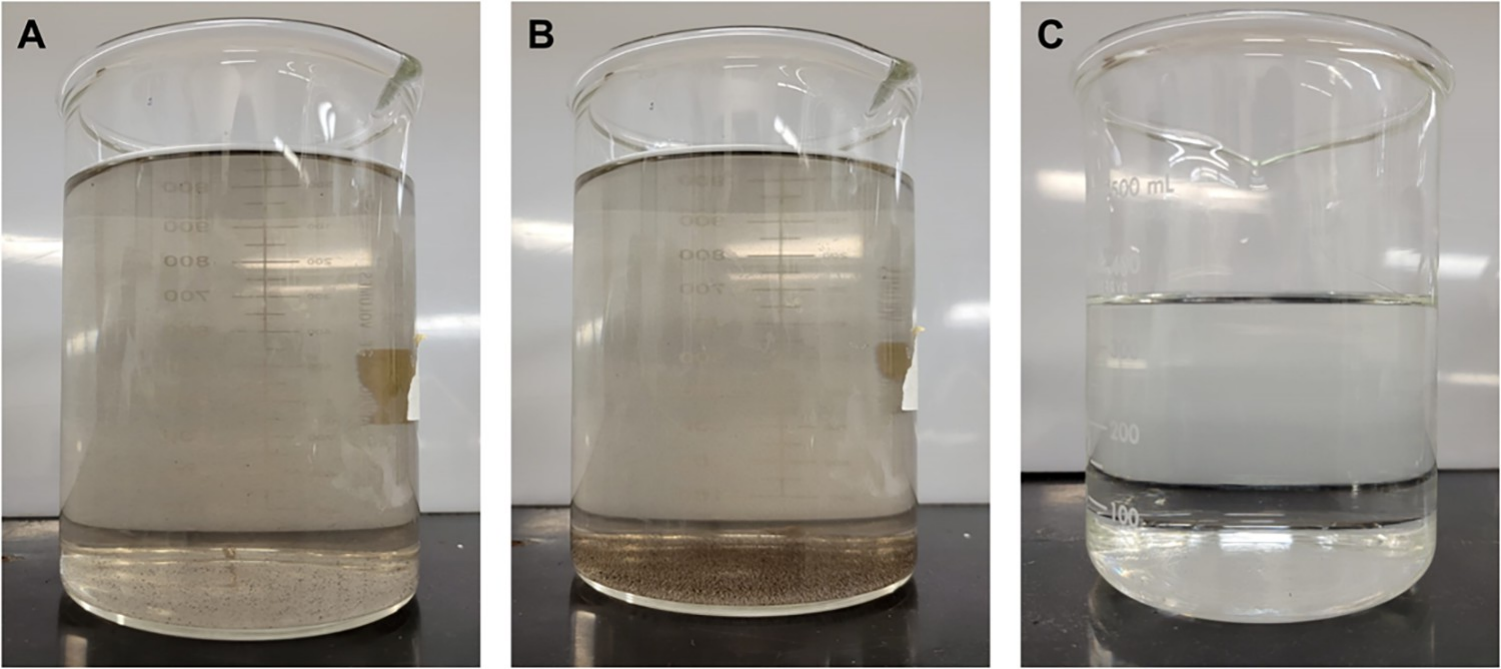
Digital images of Electro-Clean solutions after electrolysis. (A) Freshly generated HOCl solution, (B) produced HOCl solution after 1 hour of settling, and (C) produced HOCl solution after filtering through two coffee paper filters.

Wrapping a fine-mesh nylon cloth around the anode carbon rod did not hold the carbon particles within the cloth for the allotted reaction time and the solution became visibly cloudy during the electrolysis. Filtering the electrolyte post-electrolysis through two layers of ordinary coffee filter paper effectively separated the slurry particles from the electrolyte, and this filtering did not significantly affect the concentration of free chlorine. After passage through the double coffee filters, the visibly cloudy solution became visibly clear (Fig 6), and the chlorine concentration slightly decreased from its initial value of 451 ± 65 ppm as Cl_2_ to a final value of 439 ± 42 ppm.

### Effect of electrolyte pH on free chlorine production

As shown in Table 1, regardless of the initial electrolyte pH (i.e. neutral or slightly acidic), at the end of each electrolysis, the final solution pH increased to the same value of approximately 8.4. As a result, the total amount of acetic acid required for pH adjustment for a final solution with pH 6 remained about the same between the two conditions, in the range of 25 to 28 mL of acetic acid for 1.35 L electrolyzed or to-be electrolyzed salt water, as noted in Table 1. This observation is likely due to the buffering capacity of NaOH that is created during the 90-minute electrolysis. Relative to the standard deviations, only a slight difference is observed between chlorine production under slightly acidic conditions and neutral conditions, as seen in Table 2.

**Table 1:** White distilled vinegar usage for pH adjustments before and after electrolysis in acidic and neutral conditions. The values shown are the average of three trials, with the error being the standard deviation.

	Electrolysis at a near-neutral pH (~8)	Electrolysis at a slightly acidic pH (~5)
**pH before electrolysis**	8.41^(a)^ (no adjustment)	5.03 ± 0.03^(b)^ (adjusted with acetic acid)
**pH after electrolysis**	8.50 ± 0.23	8.28 ± 0.07
**pH of final HOCl solution**	5.80 ± 0.24 (adjusted with acetic acid)	5.77 ± 0.19 (adjusted with acetic acid)
**Total volume of vinegar added (mL)**	28.3 ± 1.2	24.7 ± 4.7

^(a)^The pH before electrolysis measurement was taken in the electrolyte stock solution before splitting it equally between three bottles, hence there is no standard deviation to report.

^(b)^pH adjustments were made in each of the three 1.5-L bottles, hence the standard deviation on the pH before electrolysis measurement.

**Table 2:** Chlorine production in neutral and slightly acidic electrolysis conditions. The values shown are the average of three trials, with the error being the standard deviation.

	Electrolysis at a near-neutral pH (~8)	Electrolysis at a slightly acidic pH (~5)
**Average Current (amp)**	4.87 ± 0.76	4.46 ± 0.85
**Faradaic Efficiency for Free Cl**_**2**_ **(%)**	18% ± 1.9	15% ± 1.0
**Total Cl**_**2**_ **(ppm) immediately after electrolysis (pH ~8.4)**	1297 ± 113	982 ± 177

These results suggest that regardless of a neutral or slightly acidic solution *pre-electrolysis*, pH adjustment *after electrolysis* is still needed to ensure a final pH of ~6 in the final disinfectant solution. Manufacturers of some commercially-available equipment marketed as HOCl-generators, advise the users to adjust the pH *before* electrolysis but not after, thus delivering only NaOCl [50].

With the aim for Electro-Clean to be an easy, user-friendly process, it is recommended that the electrolysis be conducted at a neutral pH and make pH adjustments only after electrolysis to minimize the number of pH adjustment steps required. Additionally, it is advised that pH adjustments be made closer to the time of use to minimize chlorine decay over time as HOCl is less stable than NaOCl.

### Free chlorine decay over time

For the non-acidified storage in both plastic and glass containers, the loss of free chlorine was less than 5% after 34 days, and the pH stayed about the same at 8.7 and 8.8, respectively. In the case of vinegar-acidified storage, the loss of free chlorine was 20% in the plastic bottle, and 16% in the glass bottle after 34 days. This is consistent with the prior literature stating that HOCl (present at pH 6) is more prone to decay than the OCl^-^ ion (present at a higher pH value) [46–48]. At the end of the 34 days, the pH levels were 5.7 for the plastic bottle and 5.9 for the glass bottle.

**Fig 7:**
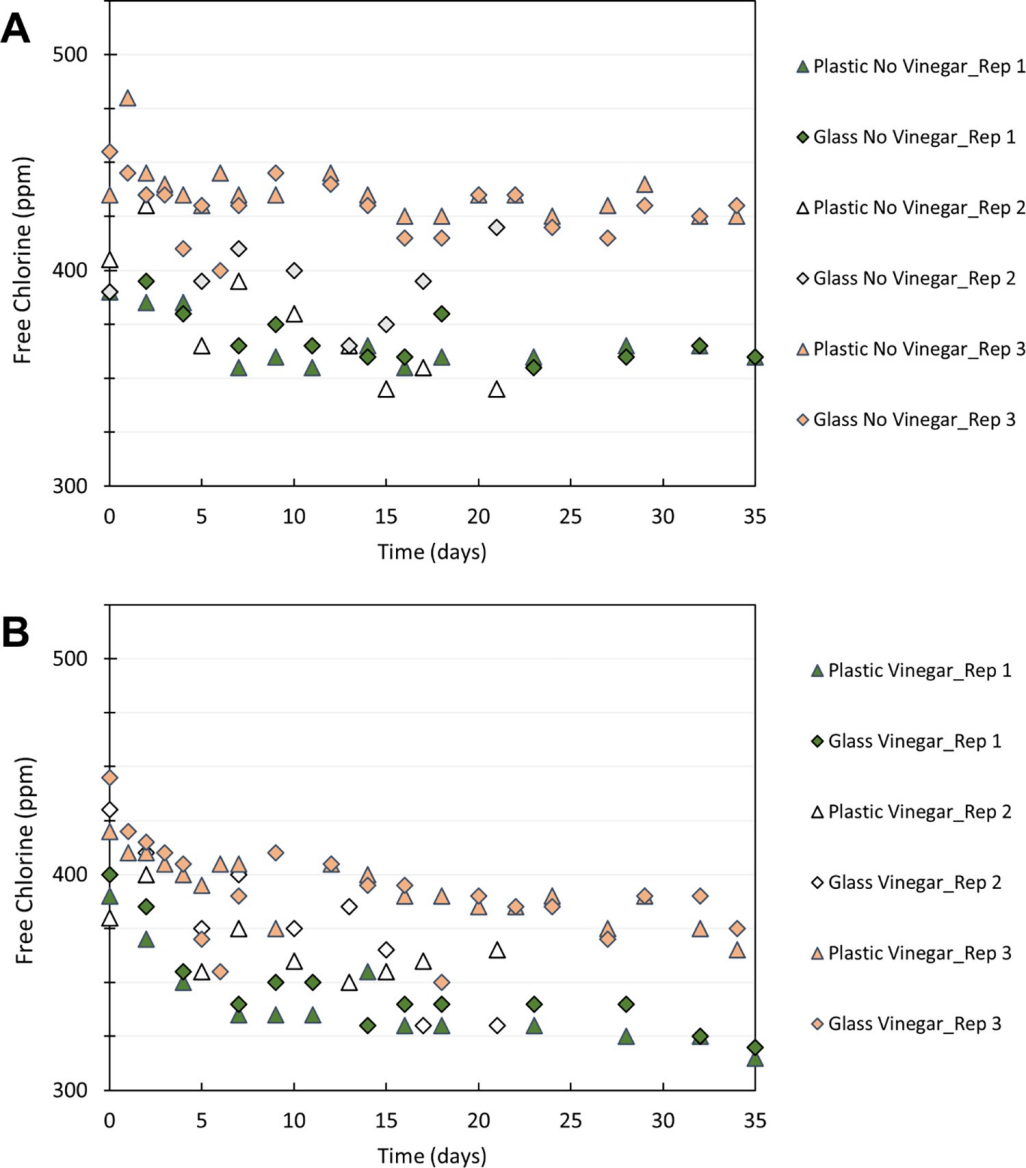
Free chlorine decay over time for stored Electro-Clean solutions. (A) Free chlorine in Electro-Clean solution without acidification, at approximately pH 8.7 stored in glass and plastic bottles, and (B) Free chlorine in Electro-Clean solution with vinegar-acidification, at approximately pH 6.4 stored in glass and plastic bottles.

As shown in Fig 7, for one month, the decay of the free chlorine concentration never exceeded 20% in the plastic or glass containers stored in a dark space. However, to maximize the disinfectant strength and maintain freshness, it is recommended that either the hypochlorous acid solution be stored in a dark, cool storage room, or that the solution’s pH be lowered only upon use.

### Microbial assays

The results of the microbial assays can be found in S5 and S6 Appendices, along with a detailed discussion of the findings. The results in Table A in S5 Appendix and Fig A in S6 Appendix suggest that the ~250 ppm hypochlorous acid solution had similar disinfecting power to bleach 1% (v/v) and ethanol 70% (v/v) on various material surfaces.

Although the literature suggests that 300 ppm of HOCl is an adequate concentration for surface disinfection, producing higher concentrations of the HOCl solution by the Electro-Clean process is recommended to ensure a disinfecting power that is comparable to ethanol 70% and bleach 1% [1]. This recommendation is based on the limited quality assurance and control of a Do-It-Yourself (DIY) product produced outside of controlled, laboratory conditions.

### Reproducibility in other communities worldwide

Co-authors and some of their collaborators within their countries, in both academic and non-academic settings, reproduced the Electro-Clean process using materials from local manufacturers and with local tap water, noting only small differences from those tested at the laboratory at UC Berkeley.

Even with these slight differences in materials, as outlined in S2 Table, co-authors produced hypochlorous acid solutions at concentrations high enough for surface disinfection (i.e., 300 ppm as HOCl), if not higher. Table 3 summarizes the free chlorine concentrations achieved byco-authors with their DIY Electro-Clean reactors before dilution and pH adjustment.

**Table 3:** Results of production of hypochlorous acid solution using Electro-Clean by co-authors in India, Mexico, Nigeria, and Uganda. Triplicates were conducted unless stated otherwise.

	Soda Bottle Scale (1.5-liter)			Community Scale (15-liter)			
	India^(a)^	Mexico	U.S.A.	India^(b)^	Nigeria^(c)^	Uganda	U.S.A.^(d)^
**Electrolysis time (min)**	64	30	90	240	240	180	90
**Volume of Electrolyte (L)**	1.0	1.3	1.35	15	15	15	15
**Average current (A)**	7.00^(e)^	4.69	4.87	16.87	4.82	7.5	6.65
**Average Free Chlorine Concentration (ppm)**	1377^(f)^	454.55	1297	500	515	640	259
**Volume of vinegar to lower pH ~6.0 (mL)**	90	11	28	75	15^(g)^	110	63^(c)^

^(a)^Results from co-author Vijay Matange.

^(b)^Results from co-authors in IIT Bombay.

^(c)^Duplicates were performed. The reported value is the average of the duplicates.

^(d)^Eight replicates were performed. The reported value represents the average.

^(e)^Higher current was likely achieved due to larger diameter (1.5-cm) rods. See S2 Table for details.

^(f)^Result estimated assuming 15% Faradaic efficiency. Free chlorine measuring kit not available.

^(g)^Hydrochloric acid (HCl) was used for pH adjustment.

### Cost analysis of disinfectant production with Electro-Clean

Tables 4 and 5 show the detailed capital and operating costs of the 1.5-liter scale Electro-Clean system. Any variability in the capital and operating costs of Electro-Clean between India, Mexico, and the USA is attributed to the relative price differences of the various components. As expected, a significant fraction (more than 50%) of capital costs for Electro-Clean are attributed to the SMPS. Cost estimates used the cost of a relatively higher amperage (5 V, 20 A) power supply; however, a lower amperage power supply can decrease the capital costs significantly. In the USA, operating costs to yield the Electro-Clean solution was $0.15 per liter at 800 ppm free chlorine concentration. The 800 ppm is representative of an average free chlorine concentration produced during the long-term experiments presented in Fig 5. Corresponding operating costs in India and Mexico are $0.09 per liter and $0.11 per liter, respectively. In India and Mexico, consumable costs of carbon rods contributed significantly (more than 50%), whereas in the USA, the operating costs are dominated by the costs of salt.

**Table 4:** Capital costs of 1.5-liter scale Electro-Clean system. Prices as of April 2023.

Item	India (Indian Rupees)	Mexico (Mexican Pesos)	United States (Dollars)
2-liter Water Bottle	77	44	2
SMPS Power Supply (5 V, 20 A)	735	400	20
Electrical wire (2x) (12 AWG, 1 meter long)	96	172	8
Multimeter	243	169	12
Total (in local currency)	1151	785	42
Total (in USD)	$14	$42	$42

**Table 5:** Operating costs of 1.5-liter scale Electro-Clean system for producing 1.35 L solution of 800 ppm free chlorine. Prices as of April 2023.

Item	India (INR per L)	Mexico (Pesos per L)	United States (USD per L)
Carbon Welding rods (2x) (~5 mm diameter, 30 cm long)	4.93	1.15	0.02
Vinegar	0.75	0.65	0.10
Sodium Chloride	1.69	0.31	0.03
Electricity	0.12	0.03	0.00
Total (in local currency per L)	7.48	2.14	0.15
Total (in USD per L)	$0.09	$0.11	$0.15

Electro-Clean is an affordable technology, with its capital costs being among the lowest for existing electrochlorinator technologies in the market for personal, household, and small-scale applications. Table 6 compares Electro-Clean with other electrochlorinator technologies, on the basis of capital cost. A comparison of operating and maintenance costs would be challenging due to most products not making that information publicly available.

**Table 6:** Capital cost comparison of available existing personal/household/small-scale electrochlorinators. Prices as of April 2023.

Product Name	Brand	Cost (USD)
Electro-Clean	Electro-Clean	42
Personal Disinfectant Generator (does not include power supply)	WaterStep	46
Force of Nature Starter Kit	Force of Nature	80
EcoOne Electrolyzed Water System	EcoloxTech	150
Commercial BleachMaker Kit	WaterStep	500

To reinforce the affordability of on-site chlorine generation compared to the purchase of ready-to-use commercial bleach products, the cost of the Electro-Clean solution was compared to the cost per liter of a commercial bleach product. When comparing these costs, it is important to note that the disinfecting properties of HOCl (the active ingredient in Electro-Clean) are much stronger than that of NaOCl (the active ingredient in bleach). Thus, greater volumes of the bleach solution of an equivalent concentration are needed to meet the same levels of disinfection. When using chlorine-based disinfectants for surface disinfection, it is typically recommended to use 200 ppm as HOCl or 2000 ppm (0.2%) as NaOCl [1, 51].

The standard price of bleach in the USA is approximately $1 USD per liter (Clorox, 7.5% as sodium hypochlorite, Amazon) [52]. When diluting the bleach stock to 0.2% for use as a surface disinfectant, the cost becomes $0.03 per liter. This can be compared to $0.04 per liter of the diluted 200 ppm HOCl solution produced by Electro-Clean (i.e., a fourth of $0.15 per liter of 800 ppm, as shown in Table 5). In Mexico, the price of bleach is approximately $0.65 USD per liter (Clorox, 7.5% as sodium hypochlorite, Mercado Libre) [53]. After dilution, the price lowers to $0.018 USD per liter. However, cost is not the only barrier to accessing disinfectants in low-resource or rural areas, as vulnerable supply chains and transportation can also play a role in accessibility. As such, comparisons in Table 6 are between Electro-Clean and other electrochlorinators, as opposed to the chlorine-based disinfectant (e.g. diluted bleach).

The low capital cost of the Electro-Clean system in comparison to other commercial alternatives, as shown in Table 6, has the potential for increasing accessibility to on-site chlorine-generating devices in low-resource settings.

### Understanding the context of intended application in communities

Collaborations were critical to the success of this project, as they allowed for rapid two-way exchange of knowledge, and allowed for sharing of findings in a variety of contexts. The production and scale-up of Electro-Clean was done in seven countries (USA, Mexico, India, Nigeria, Uganda, Mali, and Mozambique), in settings that included classrooms, households, and community organizations. Furthermore, collaboration among co-authors led to a collective awareness and understanding of the complex social and technical barriers associated with technology adoption of Electro-Clean. The availability of electrode materials, for example, varied greatly and presented challenges for selecting suitable substitutes where the recommended materials were unavailable.

Although low-cost disinfectants have always been in demand, the Electro-Clean project was initiated in response to the COVID-19 pandemic. The timeliness of these efforts is highlighted by the very rapid scale-up by co-authors at their respective institutions. For example, at Covenant University in Ota, Nigeria, co-author Omole and his students rapidly obtained permissions and administrative support for substantial scale-up efforts of Electro-Clean for practical use at the height of the COVID-19 pandemic in early 2020. Nigeria went in and out of lockdowns in 2020 as the different waves of the pandemic raged, and the demand for disinfectants soared. Thus, the advent of Electro-Clean as an affordable and safe disinfectant was a welcome contribution. The team at Covenant University responded to the sudden increased demand for surface disinfectants with a rapid scale-up of Electro-Clean, and also used the opportunity for community health promotion. They scaled up the Electro-Clean reactor design to a community scale, using 20-liter (5-gallon) plastic buckets, further described in S7 Appendix, to meet the large quantities of disinfectant in demand. This larger-scale production design of the technology, further described in Supporting Information, has great relevance for global health work since it has a greater capacity to reach and serve more users.

Over six months, the Covenant University team produced over 45 liters of the concentrated Electro-Clean disinfectant (>800 ppm as HOCl), which was diluted by adding three parts water (thus totaling 180 liters of ready-to-use disinfectant at 200 ppm as HOCl). The team distributed over five hundred 250-mL labeled spray bottles to the University faculty and students for use on high-touch surfaces in homes, classrooms, and offices. User acceptance of the disinfectant was promoted with attractive labeling of the spray bottles. The labels contained brief user instructions, a statement of regulatory approval by the University authorities, and information on where the disinfectant solution could be refilled. The contents of every spray bottle was quality controlled, with a final step of measuring the free chlorine concentration prior to distribution. Use and popularity of the high-touch surface disinfectant noticeably declined as the COVID-19 pandemic waned, airborne transmission was identified as the overwhelmingly dominant route of transmission, and people mostly returned to business as usual.

Hurdles associated with regulatory approval by local governments and public perception were encountered by the co-author and team from Takataka Plastics in Gulu, Uganda, where the on-site generated hypochlorous acid was not officially recognized as an effective and approved disinfectant. Because the new disinfectant was not approved by the country’s National Bureau of Standards, some prospective users did not believe the new disinfectant was effective.

Furthermore, the local prospective users often did not trust the chlorine-based disinfectant because it did not smell like the alcohol-based disinfectants to which they were accustomed. This challenge emerged during the scale-up of the Electro-Clean production and distribution efforts since the Uganda National Bureau of Standards only has a category for alcohol-based disinfectants and does not have tests or procedures for evaluating non-alcohol-based disinfectants [54]. To address these regulatory hurdles, that team’s future work involves developing a test protocol that could be proposed to the Bureau for testing of the chlorine-based disinfectant product for approval.

As noted earlier, COVID-19-related interest in Electro-Clean surged during the early months of the pandemic and declined as it became clear that aerosol transmission was the dominant mode of transmission. Nevertheless, the large unmet need among resource-poor communities and healthcare facilities for reliable access to surface disinfection was emphasized in a series of open virtual workshops organized by PATH (a global nonprofit dedicated to improving public health) starting in 2022 [55–57]. Establishing partnerships with hospitals and health clinics operating in rural, low-resource settings could be a next step as the demand for low-cost disinfectants will always be relevant.

## Conclusions

The development of the Electro-Clean process was not a "top-down" or "isolated" approach, but instead, a culmination of rapid design feedback from collaborators (some of them co-authors of this paper), landing on a design that made the most sense for a large fraction of intended users. As discussed in the Introduction, the selection of reactor components built off knowledge of salt water electrolysis for chlorine production, but prioritized criteria such as availability, cost, and simplicity over achieving maximum Faradaic efficiency.

Even moderately efficient chlorine production with carbon electrodes can improve public health in the face of supply shortages which create significant vulnerabilities. Although the Electro-Clean system uses commonplace welding carbon rods for electrodes, instead of expensive state-of-the-art materials, disinfectant concentrations of free chlorine (up to 1000 ppm as Cl_2_) can be produced with this approach at affordable costs. Prior to pH adjustment, the Electro-Clean process makes bleach (i.e., sodium hypochlorite, a chlorine-based disinfectant) at about five times less efficiency (Faradaic efficiency of ~20%) than off-the-shelf chlorine generator products, causing electricity costs to jump five-fold. However, electricity costs are a small fraction of the operating cost. So, a Faradaic efficiency of only ~20% (translating into 5 times more electricity use) is a great cost-saving tradeoff against the much lower cost of electrodes (graphite versus Mixed-Metal-Oxide electrodes). Because the final product is so low-cost and can be locally made, Electro-Clean is a worthwhile and accessible alternative.

In terms of biocidal activity and disinfectant performance, the hypochlorous acid solution produced by the Electro-Clean process was observed to be effective in reducing colony-forming units of bacteria on contaminated high-touch surfaces. Its effectiveness was comparable to standard disinfectant solutions (e.g. diluted bleach, and 70% ethanol). The low-cost, widely available carbon gouging electrodes used in the Electro-Clean process were shown to be reasonably durable, with a lifetime of 45 operating hours per anode and the solution concentration remained relatively stable for up to a month when properly stored in the dark and at room temperature.

Limitations of the Electro-Clean system include its need for access to electricity, and also the user training that is involved with its operation, albeit minimal. The Electro-Clean system, especially when operated in batch mode, does not require a significant amount of electricity (less than 10 watts) to generate the high-concentration disinfectant solution (up to 1,000 ppm as Cl_2_) in a short period of time (less than 2 hours). However, in contexts where access to electricity is not available, the Electro-Clean system may not be an appropriate solution. Pairing this unit with a solar system could avoid reliance on an intermittent or absent electricity supply.

Even though the Electro-Clean process is robust, effective, and low-cost, there are still inevitable challenges and social barriers to technology adoption. Through collaboration with co-authors and partners in communities around the world, it has been consistently observed that people are reluctant to adopt “complex” technologies—“the simpler the better” is often preferred. It has also been noted that implementing this technology at a household level is quite challenging, while technology adoption at a community level where one person is in charge of producing the disinfectant for other users is more well-received. To lower the barrier of entry to comfortably interact with this technology, public access to instructional documents and videos on how to replicate this work were placed online and translated into four languages as a small stride towards increasing accessibility to disinfectant solutions [58]. Public seminars and global communities of practice also exist on the topic of decentralized chlorine production [55].

By further developing this on-site HOCl production technology, using locally available materials, and enhancing outreach efforts, it is hoped that the Electro-Clean process can be a reliable solution for communities and organizations in need of a low-cost disinfectant to improve public health.

## Supporting information

S1 Appendix Faradaic efficiency theory and calculations. (DOCX)

S2 Appendix Chlorine decay in storage containers. (DOCX)

S3 Appendix Additional Electro-Clean designs. (DOCX)

S4 Appendix Long-term experiments. (DOCX)

S5 Appendix Microbiological tests in Mexico. (DOCX)

S6 Appendix Microbiological tests in India. (DOCX)

S7 Appendix Scale-up efforts by co-authors. (DOCX)

S1 Table Electrolyte composition. Water Quality Data from EBMUD, Orinda Water Treatment Plant (accessed 06 February 2023).
(DOCX)

S2 Table Specifications of the locally available materials used by co-authors in India, Mexico, Nigeria, and Uganda. (DOCX)

## References

[1] Block MS, Rowan BG. Hypochlorous Acid: A Review. J Oral Maxillofac Surg. 2020 Sep;78(9):1461–6. doi: 10.1016/j.joms.2020.06.029 32653307 PMC7315945

[2] Rationale and Considerations for Chlorine Use in Infection Control for Non- U.S. General Healthcare Settings. 2020 [cited 2023 Jan 29]. Centers for Disease Control and Prevention [Internet]. Available from: https://www.cdc.gov/vhf/ebola/clinicians/non-us-healthcare-settings/chlorine-use.html

[3] McGlynn W. Guidelines for the Use of Chlorine Bleach as a Sanitizer in Food Processing Operations. 2016 Jun[cited 2023 Jan 29]. In: Oklahoma State University—Food and Agricultural Products Research and Technology Center [Internet]; Available from: https://ucfoodsafety.ucdavis.edu/sites/g/files/dgvnsk7366/files/inline-files/26437.pdf

[4] Hand S, Cusick RD. Electrochemical Disinfection in Water and Wastewater Treatment: Identifying Impacts of Water Quality and Operating Conditions on Performance. Environ Sci Technol. 2021 Mar 16;55(6):3470–82. doi: 10.1021/acs.est.0c06254 33616403 PMC7970539

[5] Lindmark M, Cherukumilli K, Crider YS, Marcenac P, Lozier M, Voth-Gaeddert L, et al. Passive In-Line Chlorination for Drinking Water Disinfection: A Critical Review. Environ Sci Technol. 2022 Jul 5;56(13):9164–81. doi: 10.1021/acs.est.1c08580 35700262 PMC9261193

[6] Drolet A. Global Community of Practice (CoP) on decentralized chlorine production. PATH. [Quaterly Meeting]. 2021 Aug.

[7] Percival SL, Yates MV, Williams DW, Chalmers RM, Gray NF. Microbiology of Waterborne Diseases. 2nd ed. Elsevier; 2014.

[8] Hakim H, Thammakarn C, Suguro A, Ishida Y, Kawamura A, Tamura M, et al. Evaluation of sprayed hypochlorous acid solutions for their virucidal activity against avian influenza virus through in vitro experiments. J Vet Med Sci. 2015 Feb;77(2):211–5. doi: 10.1292/jvms.14-0413 25421399 PMC4363024

[9] Nguyen K, Bui D, Hashemi M, Hocking DM, Mendis P, Strugnell RA, et al. The Potential Use of Hypochlorous Acid and a Smart Prefabricated Sanitising Chamber to Reduce Occupation-Related COVID-19 Exposure. Risk Manag Healthc Policy. 2021 Jan 22;14:247–52. doi: 10.2147/RMHP.S284897 33519249 PMC7837568

[10] Rasmussen ED, Williams JF. Stabilized hypochlorous acid disinfection for highly vulnerable populations: Brio HOCL wound disinfection and area decontamination. 2017. [cited 2023 Nov 12]. p. 1–8. In: 2017 IEEE Global Humanitarian Technology Conference (GHTC) [Internet]. Available from: https://ieeexplore.ieee.org/document/8239259

[11] Anagnostopoulos AG, Rong A, Miller D, Tran A, Head T, Lee MC, et al. 0.01% Hypochlorous Acid as an Alternative Skin Antiseptic: An In Vitro Comparison. Dermatol Surg. 2018 Dec;44(12):1489–93. doi: 10.1097/DSS.0000000000001594 29985866

[12] Kubota A, Goda T, Tsuru T, Yonekura T, Yagi M, Kawahara H, et al. Efficacy and safety of strong acid electrolyzed water for peritoneal lavage to prevent surgical site infection in patients with perforated appendicitis. Surg Today. 2015 Jul 1;45(7):876–9. doi: 10.1007/s00595-014-1050-x 25387655

[13] Fam A, Finger PT, Tomar AS, Garg G, Chin KJ. Hypochlorous acid antiseptic washout improves patient comfort after intravitreal injection: A patient reported outcomes study. Indian J Ophthalmol. 2020 Nov;68(11):2439. doi: 10.4103/ijo.IJO_2001_20 33120635 PMC7774204

[14] Wang L, Bassiri M, Najafi R, Najafi K, Yang J, Khosrovi B, et al. Hypochlorous Acid as a Potential Wound Care Agent. J Burns Wounds. 2007 Apr 11;6:e5. 17492050 PMC1853323

[15] Overholt B, Reynolds K, Wheeler D. 1151. A Safer, More Effective Method for Cleaning and Disinfecting GI Endoscopic Procedure Rooms. Open Forum Infect Dis. 2018 Nov 26;5(Suppl 1):S346.

[16] Kott Y, Nupen EM, Ross WR. The effect of pH on the efficiency of chlorine disinfection and virus enumeration. Water Research. 1975 Oct 1;9(10):869–72.

[17] Benjamin MM. Water Chemistry. Waveland Press; Vol. 2nd Edition. 2015.

[18] Electrolytic Cells. [cited 2023 Jan 29]. In: Bodner Research Web [Internet]. Available from: http://chemed.chem.purdue.edu/genchem/topicreview/bp/ch20/faraday.php

[19] Wang Y, Liu Y, Wiley D, Zhao S, Tang Z. Recent advances in electrocatalytic chloride oxidation for chlorine gas production. J Mater Chem A. 2021 Sep 14;9(35):18974–93.

[20] Ejaz T, Saadia S, Akhlaq S, Aziz A, Ahmed MA, Siddiqui AF. Clinical Features and Outcomes of Acute Chlorine Gas Inhalation; a Brief Report. Arch Acad Emerg Med. 2022 Feb 14;10(1). doi: 10.22037/aaem.v10i1.1448 35402997 PMC8986491

[21] Morim A, Guldner GT. Chlorine Gas Toxicity. Treasure Island (FL): StatPearls Publishing; 2023 [cited 2023 Nov 15]. In: StatPearls [Internet]. Available from: http://www.ncbi.nlm.nih.gov/books/NBK537213/30725898

[22] Choi J, Shim S, Yoon J. Design and operating parameters affecting an electrochlorination system. J Ind Eng Chem. 2013 Jan 25;19(1):215–9.

[23] Abdul-Wahab SA, Al-Weshahi MA. Brine Management: Substituting Chlorine with On-Site Produced Sodium Hypochlorite for Environmentally Improved Desalination Processes. Water Resour Manag. 2009 Sep 1;23(12):2437–54.

[24] Saha J, Gupta SK. A novel electro-chlorinator using low cost graphite electrode for drinking water disinfection. Ionics. 2017 Jul 1;23(7):1903–13.

[25] Green Cleaning Products for Commercial Use. [cited 2023 Nov 15]. In: GenEon Technologies [Internet]. Available from: https://www.geneontechnologies.com/our-products/

[26] Shop at Force of Nature. [cited 2023 Nov 15]. In: Force of Nature [Internet]. Available from: https://www.forceofnatureclean.com/shop/

[27] All Ecoloxtech HOCL Systems. [cited 2023 Nov 15]. In: Ecoloxtech [Internet]. Available from: https://ecoloxtech.com/collections/all-hocl-systems-1

[28] Snowdon MR, Rathod S, Fattahi A, Khan A, Bragg LM, Liang R, et al. Water Purification and Electrochemical Oxidation: Meeting Different Targets with BDD and MMO Anodes. Environments. 2022 Nov;9(11):135.

[29] Hubert MA, King LA, Jaramillo TF. Evaluating the Case for Reduced Precious Metal Catalysts in Proton Exchange Membrane Electrolyzers. ACS Energy Lett. 2022 Jan 14;7(1):17–23.

[30] Choi S, Choi WI, Lee JS, Lee CH, Balamurugan M, Schwarz AD, et al. A Reflection on Sustainable Anode Materials for Electrochemical Chloride Oxidation. Adv Mater. 2023 Oct;35(43):2300429. doi: 10.1002/adma.202300429 36897816

[31] Adjodah D, Kwan J, Lichtblau S, Talsma A. Chlorine Generation: The Do-it-Yourself Approach. 2021 Mar 25 [cited 2023 Nov 15]. In: Silo.Tips [Internet]. Available from: https://silo.tips/download/chlorine-generation-the-do-it-yourself-approach#

[32] Rogers EM, Singhal A, Quinlan MM. Diffusion of Innovations. In: An Integrated Approach to Communication Theory and Research. 2nd ed. Routledge; 2008.

[33] Chemical Disinfectants | Disinfection & Sterilization Guideline. 2023 Nov 28 [cited 2024 May 21]. In: Centers for Disease Control and Prevention [Internet]. Available from: https://www.cdc.gov/infection-control/hcp/disinfection-sterilization/chemical-disinfectants.html?CDC_AAref_Val=https://www.cdc.gov/infectioncontrol/guidelines/disinfection/disinfection-methods/chemical.html

[34] Cleaning and disinfection of environmental surfaces in the context of COVID-19. 2020 May 16 [cited 2022 Mar 3]. In: World Health Organization [Internet]. Available from: https://www.who.int/publications-detail-redirect/cleaning-and-disinfection-of-environmental-surfaces-inthe-context-of-covid-19

[35] US EPA O. About List N: Disinfectants for Coronavirus (COVID-19). 2020 [cited 2022 Mar 3]. In: U.S. Environmental Protection Agency [Internet]. Available from: https://www.epa.gov/coronavirus/about-list-n-disinfectants-coronavirus-covid-19-0

[36] Water quality data. [cited 2023 Jan 29]. In: East Bay Municipal Utility District [Internet]. Available from: https://www.ebmud.com/water/about-your-water/water-quality/water-quality-data

[37] Deborde M, von Gunten U. Reactions of chlorine with inorganic and organic compounds during water treatment—Kinetics and mechanisms: A critical review. Water Res. 2008 Jan 1;42(1):13–51. doi: 10.1016/j.watres.2007.07.025 17915284

[38] Westerhoff P, Chao P, Mash H. Reactivity of natural organic matter with aqueous chlorine and bromine. Water Res. 2004 Mar 1;38(6):1502–13. doi: 10.1016/j.watres.2003.12.014 15016527

[39] Rabah MA, Nassif N, Azim AAA. Electrochemical wear of graphite anodes during electrolysis of brine. Carbon. 1991 Jan 1;29(2):165–71.

[40] Hine F, Yasuda M, Sugiura I, Noda T. Effects of the Active Chlorine and the pH on Consumption of Graphite Anode in Chlor‐Alkali Cells. J Electrochem Soc. 1974 Feb 1;121(2):220.

[41] Entwisle JH. Consumption of graphite anodes in chlorine manufacture by brine electrolysis. J Appl Electrochem. 1974 Nov 1;4(4):293–303.

[42] Ryan MP, Williams DE, Chater RJ, Hutton BM, McPhail DS. Why stainless steel corrodes. Nature. 2002 Feb;415(6873):770–4. doi: 10.1038/415770a 11845203

[43] Ibrahim MAM, Abd El Rehim SS, Hamza MM. Corrosion behavior of some austenitic stainless steels in chloride environments. Mater Chem Phys. 2009 May 15;115(1):80–5.

[44] Freire L, Carmezim MJ, Ferreira MGS, Montemor MF. The electrochemical behaviour of stainless steel AISI 304 in alkaline solutions with different pH in the presence of chlorides. Electrochimica Acta. 2011 May 30;56(14):5280–9.

[45] Janssen LJJ, Hoogland JG. Electrolysis of acidic NaCl solution with a graphite anode—I. The graphite electrode. Electrochimica Acta. 1969 Nov 1;14(11):1097–108.

[46] Lister MW. The decomposition of hypochlorous acid. Can J Chem. 1952 Nov;30(11):879–89.

[47] Ishihara M, Murakami K, Fukuda K, Nakamura S, Kuwabara M, Hattori H, et al. Stability of Weakly Acidic Hypochlorous Acid Solution with Microbicidal Activity. Biocontrol Sci. 2017;22(4):223–7. doi: 10.4265/bio.22.223 29279579

[48] Adam LC, Fabian I, Suzuki K, Gordon G. Hypochlorous acid decomposition in the pH 5–8 region. Inorg Chem. 1992 Aug 1;31(17):3534–41.

[49] Hakim H, Alam MS, Sangsriratanakul N, Nakajima K, Kitazawa M, Ota M, et al. Inactivation of bacteria on surfaces by sprayed slightly acidic hypochlorous acid water: in vitro experiments. J Vet Med Sci. 2016 Jul;78(7):1123–8. doi: 10.1292/jvms.16-0075 27052464 PMC4976267

[50] J M. Electrolyzed Water 101. 2023 [cited 2023 Nov 12]. In: Force of Nature [Internet]. Available from: https://www.forceofnatureclean.com/electrolyzed-water-101/

[51] Bleach Dilution Ratio for Disinfecting. 2021 [cited 2023 May 4]. In: Clorox [Internet]. Available from: https://www.clorox.com/learn/bleach-dilution-ratio-chart/

[52] Clorox: Laundry & Bleach. [cited 2023 May 4]. In: Amazon.com [Internet]. Available from: https://www.amazon.com/stores/Clorox/LaundryBleach/page/2E893A5C-7B65-43EB-A741-A140B67F07C4

[53] Blanqueador Clorox Triple Acción Original 5,8 Lt—$ 51.07 [cited 2023 May 4]. In: Mercado Libre [Internet]. Available from: https://articulo.mercadolibre.com.mx/MLM-1868021657-blanqueador-clorox-triple-accion-original-58-lt-_JM

[54] Uganda National Bureau Of Standards. [cited 2023 Nov 15]. In: Uganda National Bureau Of Standards [Internet]. Available from: https://www.unbs.go.ug//content.php?src=what-is-testing?&content

[55] PATH launches Global Community of Practice on decentralized chlorine production. [cited 2023 Jan 29]. In: DefeatDD [Internet]. Available from: https://www.defeatdd.org/blog/path-launches-global-community-practice-decentralized-chlorine-production

[56] Duvernay PG, de Laguiche E, Campos Nogueira R, Graz B, Nana L, Ouédraogo W, et al. Preventing nosocomial infections in resource-limited settings: An interventional approach in healthcare facilities in Burkina Faso. Infect Dis Health. 2020 Aug 1;25(3):186–93. doi: 10.1016/j.idh.2020.04.003 32417112 PMC7211687

[57] Nogueira RC, Nigro M, Veuthey J, Tigalbaye C, Bazirutwabo B, Thior MD, et al. Can Locally Produced Chlorine Improve Water Sanitation & Hygiene (WASH) Indicators in Health Care Facilities (HCF) in Rural Chad?. Health Sciences and Disease. 2021 Nov 1;22(11).

[58] Electro-Clean. [cited 2023 Jan 29]. In: Gadgil Lab for Energy & Water Research | UC Berkeley [Internet]. Available from: https://gadgillab.berkeley.edu/electro-clean/

