# Supplementary material for: Low-cost, local production of a safe and effective disinfectant for resource-constrained communities
Source: PLOS Glob Public Health. 2024 Jun 25;4(6):e0002213. doi: 10.1371/journal.pgph.0002213 (PMC11198905; doi:10.1371/journal.pgph.0002213)
Supplement: S1 Appendix — (DOCX) [file pgph.0002213.s001.docx]

# **S1 Appendix. Faradaic Efficiency Theory and Calculations.**

Faraday’s Law explains chlorine production via electrolysis being proportional to the amount of electrical current applied. Faraday’s constant (96,485 C/mol-electron) represents the amount of electrical charge (Coulombs) contained in a mole of electrons. Two mole-electrons are needed to release one molecule of Cl_2_. By Faraday’s law, 2 x 96,485 Coulombs will release 1 mol of Cl_2_, where one mol of Cl_2_ weighs 71 grams. At 100% Faradaic efficiency, approximately 193 Coulombs release 71 milligrams of Cl_2_. The measured chlorine produced is compared to the theoretical value to represent the actual faradaic efficiency of the graphite-rod electrodes. A
