# Supplementary material for: Low-cost, local production of a safe and effective disinfectant for resource-constrained communities
Source: PLOS Glob Public Health. 2024 Jun 25;4(6):e0002213. doi: 10.1371/journal.pgph.0002213 (PMC11198905; doi:10.1371/journal.pgph.0002213)
Supplement: S2 Appendix — (DOCX) [file pgph.0002213.s002.docx]

**S2 Appendix. Chlorine decay in storage containers.**


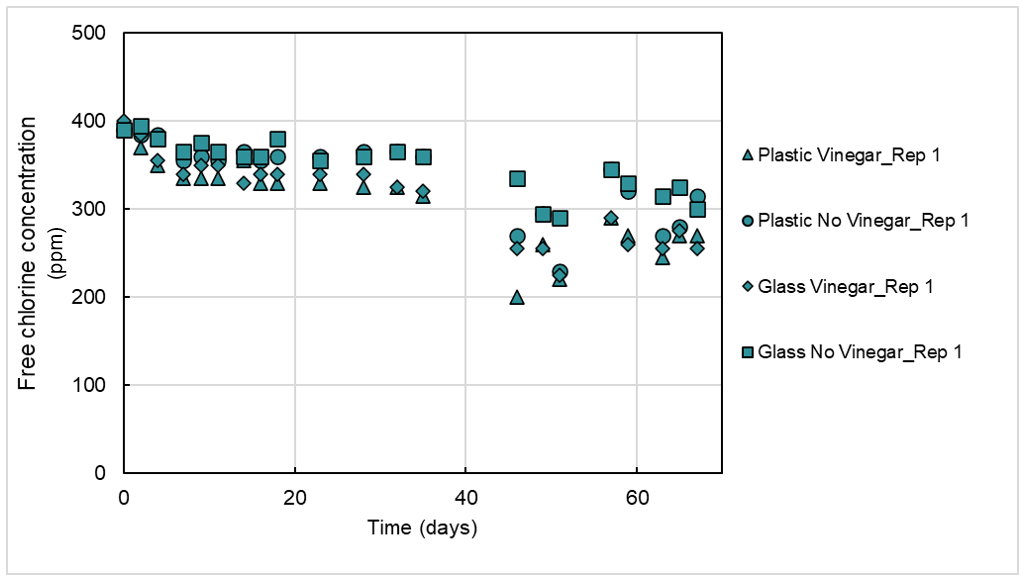


**Fig A. Free chlorine decay over time (~ 2 months) for produced HOCl solution stored without acidification and with vinegar-acidification, in glass or plastic containers.**

**Table A.** **Decay of Free Chlorine in Stored HOCl Solution.**

| **Experiment** | **Percent Removal (Cl_2_)** | **Final concentration Cl_2_ (ppm)** | **Initial pH** | **Final pH after 34 days** |
| --- | --- | --- | --- | --- |
| Plastic Bottle - Vinegar | 19.8% | 365 | 6.25 | 5.67 |
| Plastic Bottle - No Vinegar | 2.3% | 425 | 8.79 | 8.72 |
| Glass Bottle - Vinegar | 15.7% | 375 | 6.49 | 5.91 |
| Glass Bottle - No Vinegar | 5.5% | 430 | 8.79 | 8.75 |
