# Supplementary material for: Low-cost, local production of a safe and effective disinfectant for resource-constrained communities
Source: PLOS Glob Public Health. 2024 Jun 25;4(6):e0002213. doi: 10.1371/journal.pgph.0002213 (PMC11198905; doi:10.1371/journal.pgph.0002213)
Supplement: S3 Appendix — (DOCX) [file pgph.0002213.s003.docx]

**S3 Appendix. Additional Electro-Clean designs.**

**Household Scale**

***Carbon rod anode with stainless steel cathode***

A household scale reactor was configured using a glass 250-mL beaker with a stainless steel spoon as the cathode and a carbon rod as the anode. The carbon rod was extracted from a Panasonic Carbon Zinc D-cell battery using large pliers to peel away the flexible metal casing. The carbon rod was washed with mild soap and water for a few minutes to remove the excess manganese that is present in the battery. A DC USB step-up/down power supply regulator module 3.5V-12V to 1.0V-24V (brand PEMENOL) was used to provide a constant potential of 5 Volts.

The first set of experiments was conducted in 30,000 mg/L NaCl (Morton iodized salt) dissolved in Milli-Q water. The second set of experiments was conducted in synthetic tap water. The tap water composition is based on the East Bay Municipal Utility District’s water quality report. This allowed an additional complexity within the solution to achieve real-world representative conditions while still controlling for the present ions and their concentrations.

***Chlorine generation of household scale***

In a 250-mL cup with two carbon rods extracted from carbon-zinc batteries serving as electrodes and an electrolyte of 30,000 mg/L NaCl in MQ water, about 600 ppm of free chlorine was generated in about 30 minutes. In synthetic tap water, about 250 ppm of free chlorine was generated in only 10 minutes and up to 1,000 ppm of free chlorine in 60 minutes. In all cases, the target of 200 ppm of free chlorine was surpassed, as shown in Fig A.


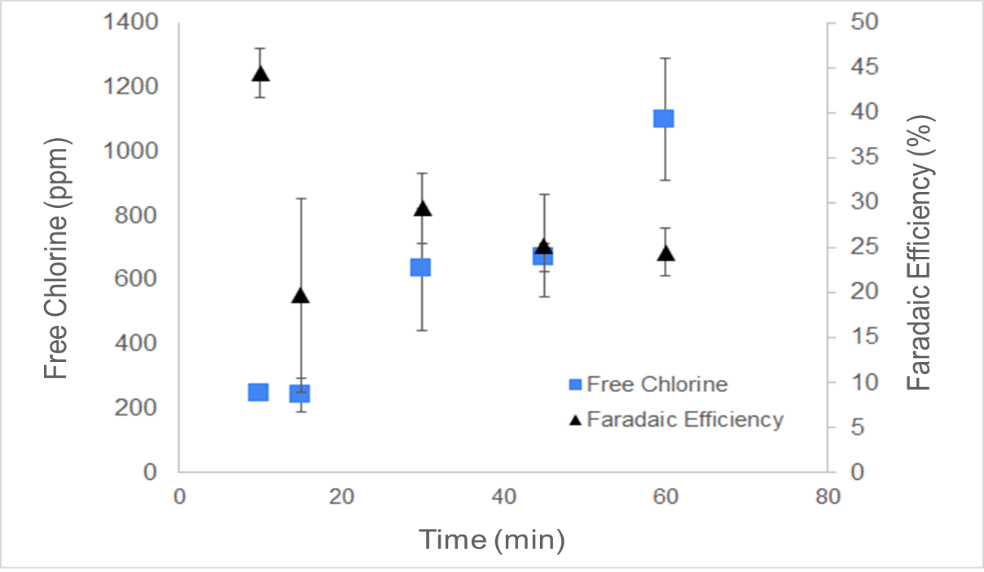


**Fig A. Free chlorine generated with carbon rod electrodes in synthetic tap water and Faradaic Efficiency at increasing experiment durations.**

**Community Scale**

***Bare carbon welding rods***

A 5-gallon, cylindrical HDPE bucket served as the reaction vessel. Four bare carbon welding rods with 1 cm diameter and 30 cm in length served as the anode. A 35 cm by 35 cm stainless steel plate with 2 mm thickness served as the cathode. The stainless steel plate was bent so that it could fit inside the bucket. To prevent contact between the electrodes, two pieces of plastic mesh were placed on the stainless steel plate, as illustrated in Fig B. The bucket lid was used to hold the four bare carbon welding rods in place. Six holes, with 1-cm diameter, were drilled on the lid of the bucket, as shown in Fig C. A 5V DC, 40-Amp capacity Switched-mode Power Supply (SMPS) served as the power supply. Nickel-steel, fully insulated alligator clips with 2.5 cm jaw openings were used to connect the electrodes to the power supply. In the case of the four carbon welding rods, each rod had an alligator clip. The loose ends of the alligator clip cables from each rod were connected to a 30 cm x 2.5 cm piece of copper metal. The cable that came from the positive terminal of the SMPS was also connected to this piece of copper metal. A clamp meter (Fluke 376 TRUE RMS CLAMP METER) was used to measure the current passing through the system.


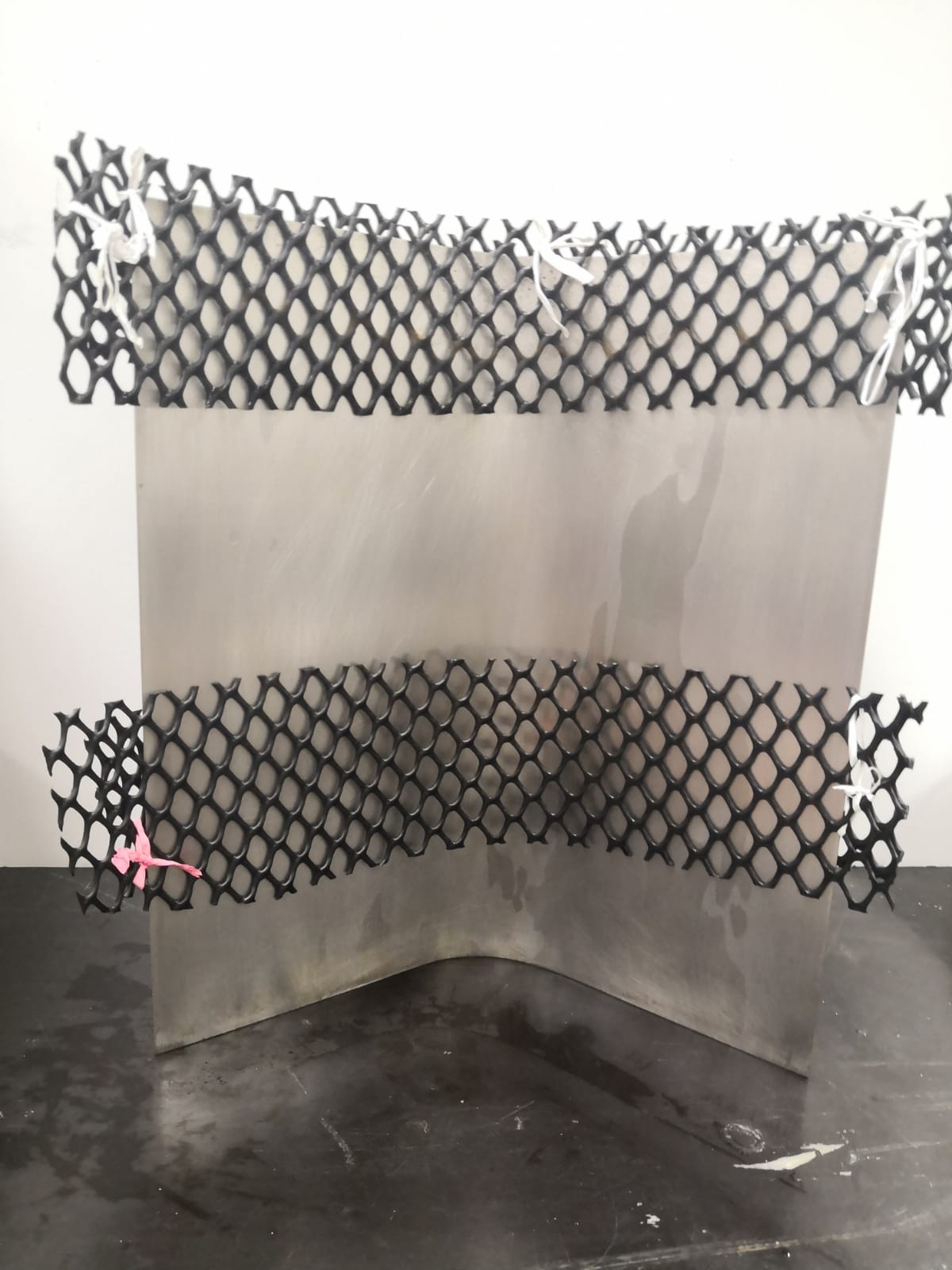

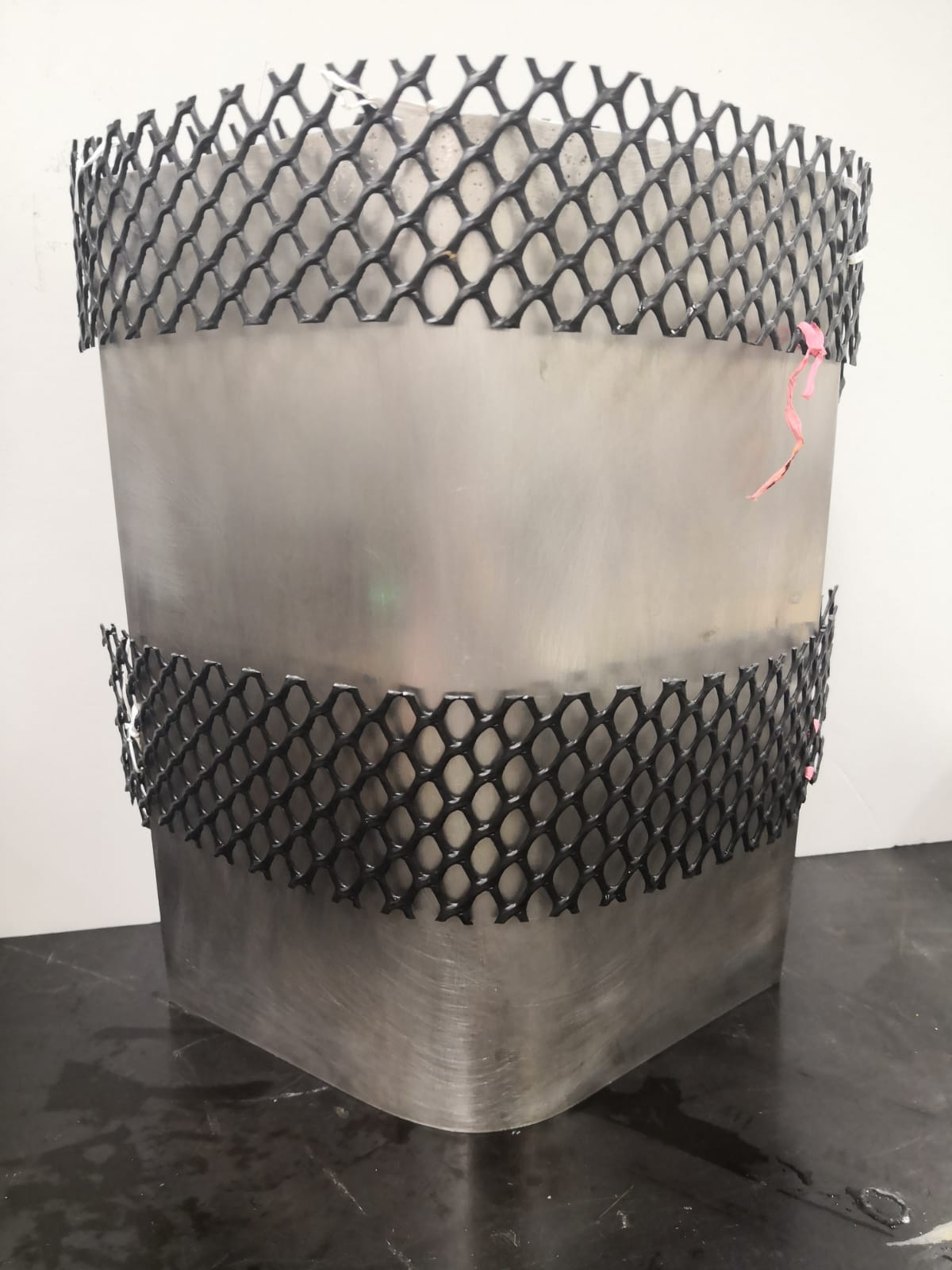


**A**

**B**

**Fig B. Digital images of stainless steel plate with two pieces of protective plastic mesh.** (A) Front view of stainless steel plate with pieces of plastic mesh. (B) Back view of stainless steel plate with pieces of plastic mesh. The two plastic mesh pieces facing each other were secured using plumber’s tape. Digital images taken by Andrea Naranjo-Soledad.

**Fig C. Illustration of the plastic lid with 6 holes drilled with a 1-cm drill tip.** The red circles represent the holes where the carbon welding rods (anode) go and the orange circle represents the hole where the alligator clip connecting the stainless steel plate (cathode) goes. The black circle represents the hole for preventing pressure buildup.

***Chlorine generation and effect of poor connections***

Chlorine gas generated during electrolysis can oxidize the teeth of the alligator clips if these are not properly insulated with dielectric grease. The rusting of the alligator clips increases the resistance in the connection between the rod and the alligator clip. Fig D shows how the free chlorine concentration decreased in a linear fashion, while the Faradaic efficiency remained constant. This indicated that the decrease in free chlorine in the system was not due to passivation, as passivation would have lowered the Faradaic efficiency. After noticing this trend, the rusted alligator clips were replaced by new, fully insulated alligator clips. The free chlorine generated increased after changing the rusted alligator clips with new ones, while the Faradaic efficiency remained relatively constant.


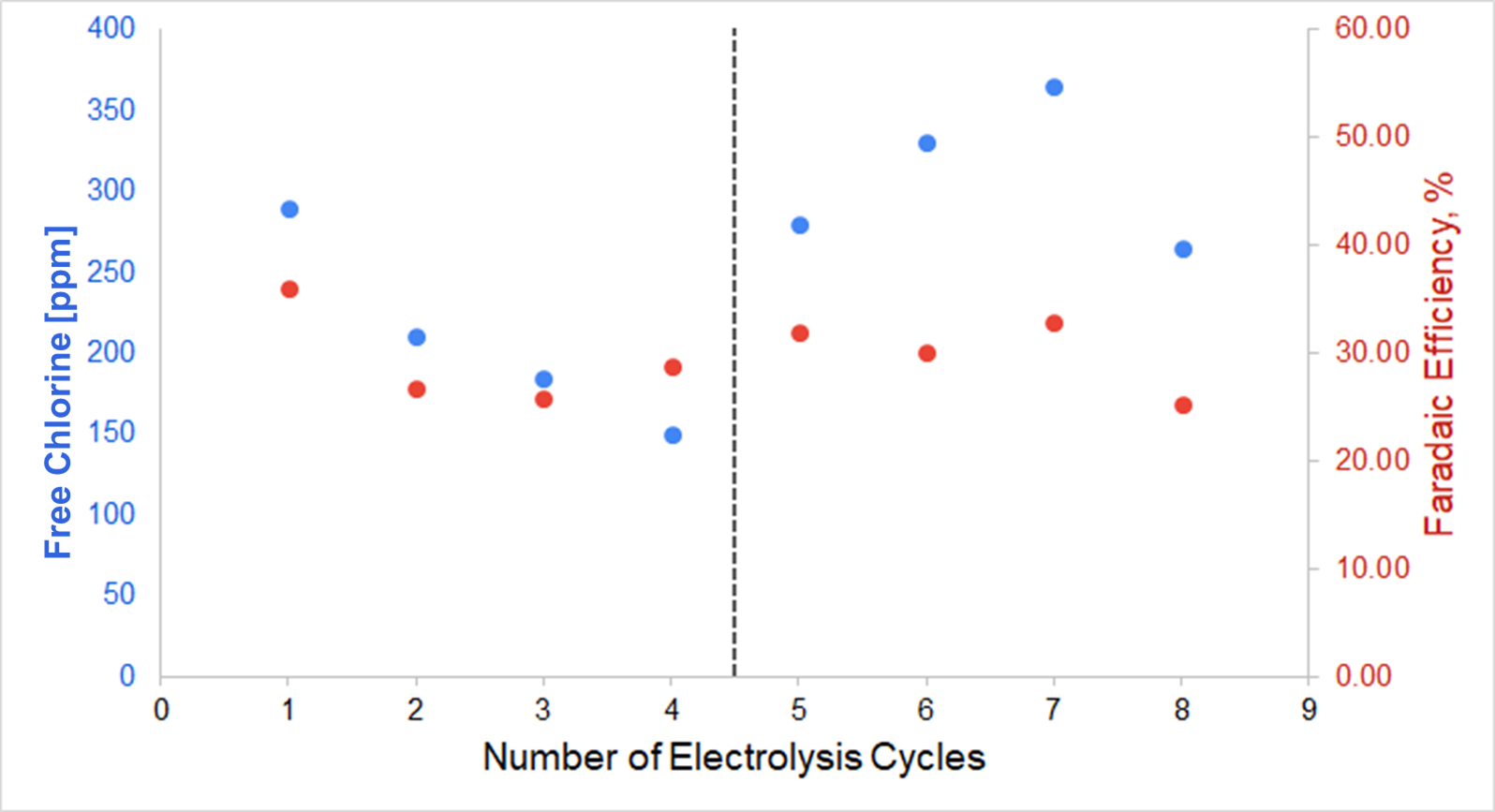


**Fig D. Free chlorine concentration versus the number of electrolysis cycles.** Each electrolysis cycle consisted of 90 minutes of electrolysis before cleaning the electrodes with a brush and water, and a fresh batch of 30,000 ppm of NaCl in synthetic tap water. The black vertical dashed line shows the before and after adding new, fully insulated, nickel-steel alligator clips.

**Co-Axial Reactor Design**

Electro-Clean devices with carbon rods inside a cylindrical pipe were investigated to understand HOCl production with a co-axial carbon rod as an anode and different cathode materials (carbon rod versus copper pipe). These experiments were conducted under constant voltage (5 V DC), having an electrolyte composition of 30,000 mg/L NaCl in tap water.

In the first configuration of co-axial electrodes, two carbon rods (~1 cm diameter, 27 cm long each) were used as electrodes. These rods were separated by a ~0.3 cm thick plastic mesh and inserted into a 1.9 cm-diameter PVC plastic pipe. The active anode and cathode surface area of the electrodes was 85 cm^2^. The electrolyte was continuously pumped into the plastic pipe with the carbon rods in up-flow mode at a flow rate of 70 mL/min using a peristaltic pump. Herein this system is referred to as “carbon rods in plastic pipe assembly” and is shown in Fig E(A).

In the second system, a carbon rod of 1 cm diameter and 27 cm length is placed at the center of a copper pipe (2.54 cm diameter). The carbon rod acted as the anode and the copper pipe acted as the cathode. The active anode and cathode surface area of the electrodes in this system were 85 cm^2^ and 215 cm^2^, respectively. Using a peristaltic pump, the electrolyte was pumped into the copper pipe-carbon rod assembly at a rate of 70 mL/min in up-flow mode. This system is referred to as “carbon rod in copper pipe assembly” and it is shown in Fig E(B).


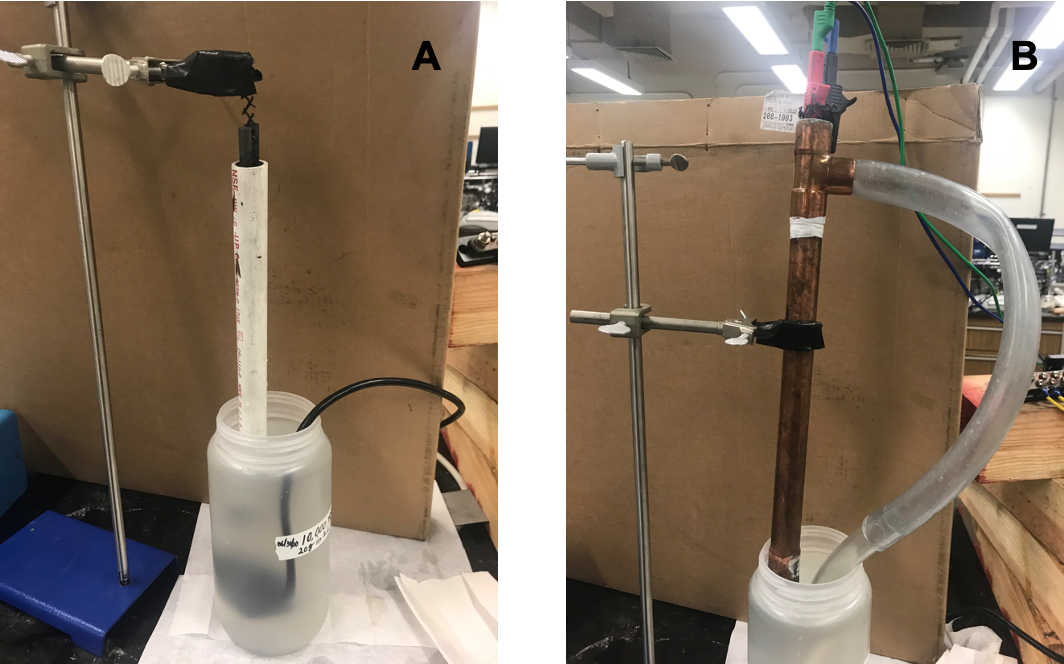


**Fig E. Digital images of the co-axial designs.** (A) Co-axial design with carbon rods in plastic pipe assembly, and (B) Co-axial design with carbon rod in copper pipe assembly. Digital pictures taken by Siva RS Bandaru

Fig F(A) shows the HOCl concentrations obtained with “carbon rods in plastic pipe” and “carbon rods in copper pipe” at different electrolysis times when operated in batch mode (electrolyte was recirculated during the experiment). Both systems achieved an average HOCl concentration in excess of 1000 ppm as Cl_2_ after 20 minutes of electrolysis. Nearly 20% higher HOCl concentration was obtained with the “carbon rod in copper pipe” system than the “carbon rods in plastic pipe” system.

In long-term experiments, the HOCl concentration reached below the target value of 200 ppm after 25 hours and 55 hours of continuous operation in “carbon rods in plastic pipe” and “carbon rods in copper pipe” systems, respectively. Significant differences in the cathode surface areas of 85 cm^2^ in “carbon rods in plastic pipe” and of 215 cm^2^ in “carbon rods in copper pipe” resulted in proportional HOCl concentrations. Before the failure points (HOCl concentration less than 200 ppm), the average HOCl concentrations in these two systems were 210 ± 5 ppm and 456 ± 60 ppm, respectively. The Faradaic efficiency of HOCl production remained constant during 70 hours of operation.


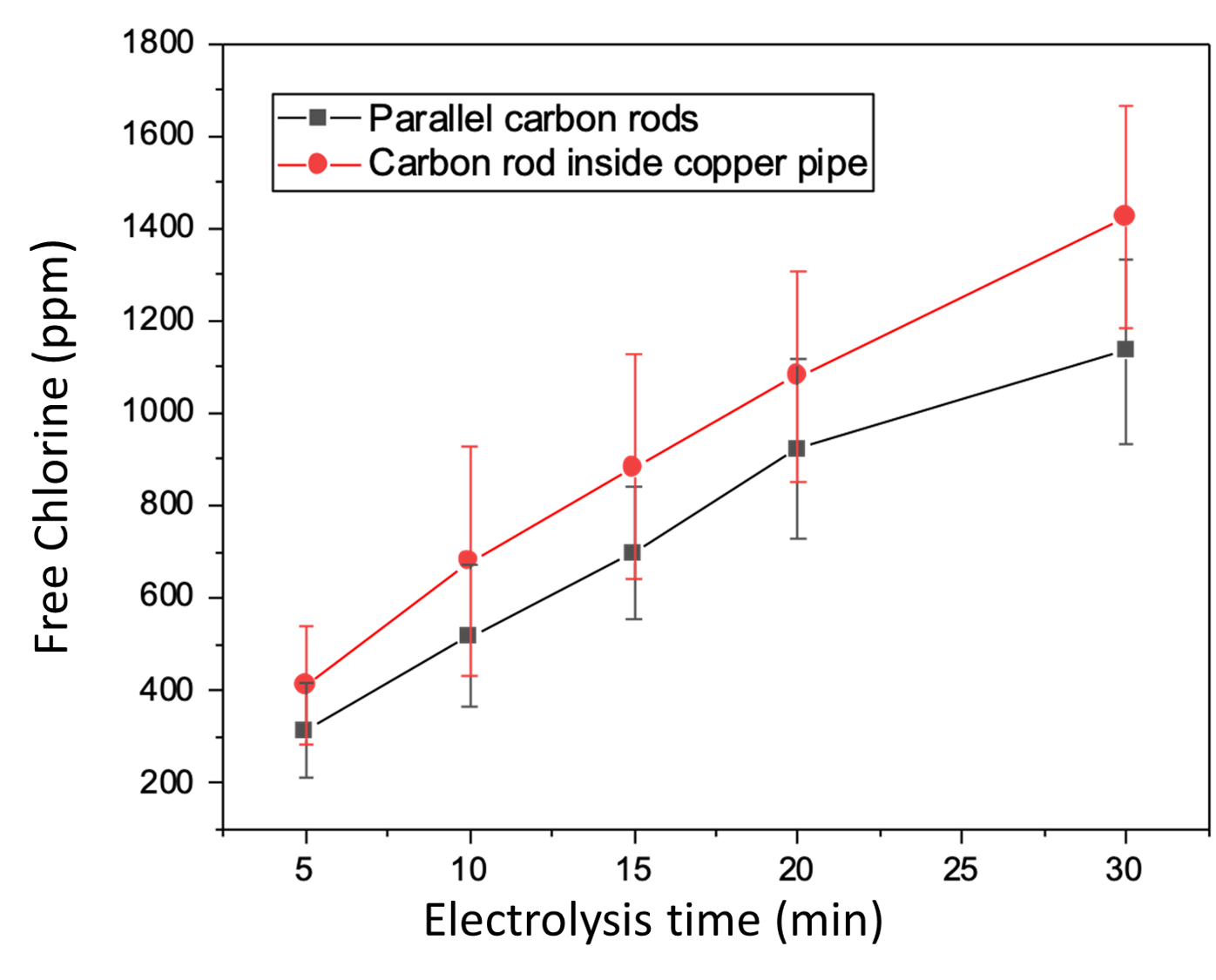


**A**


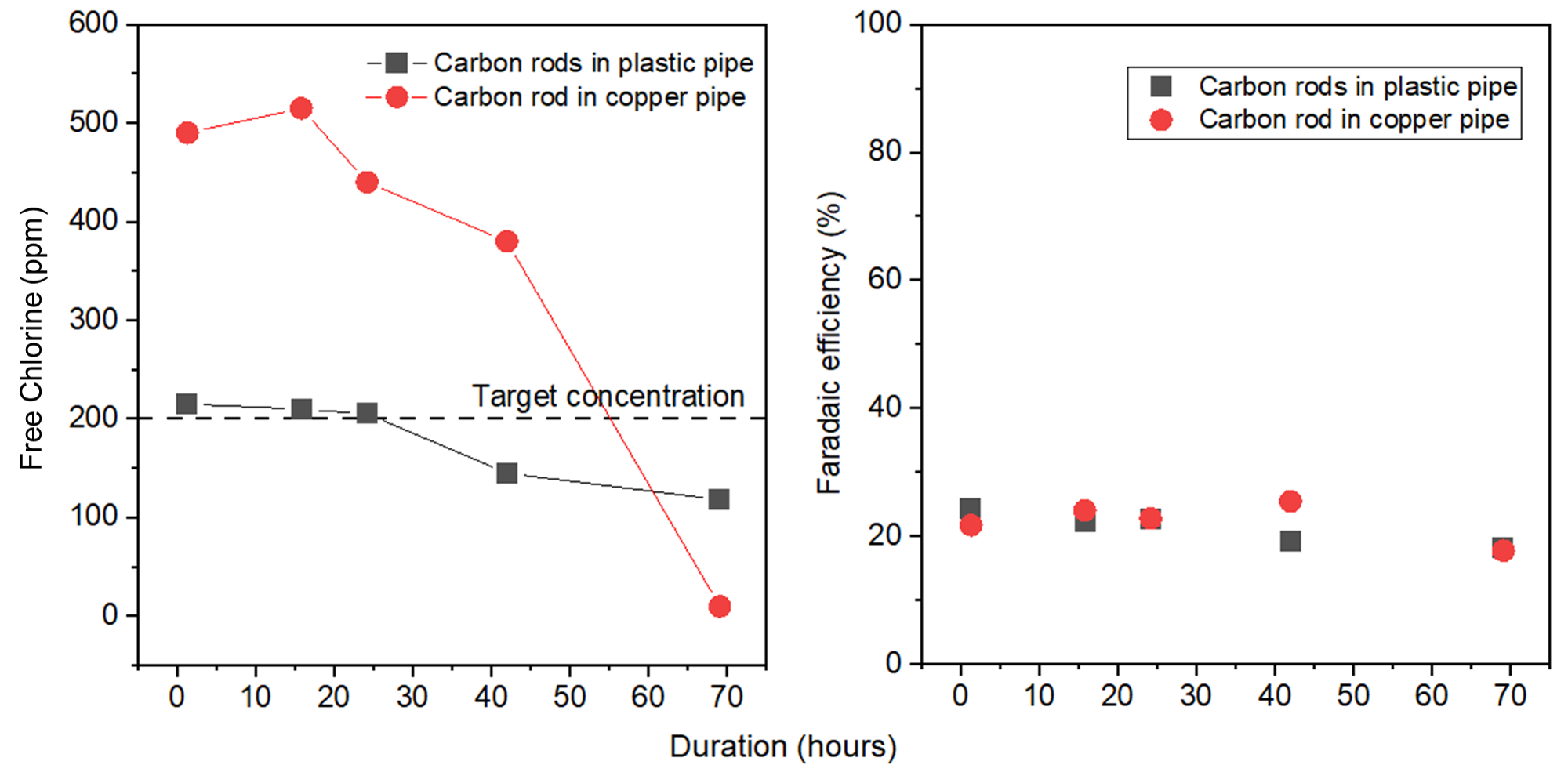


**B**

**Fig F. Co-axial systems operated in batch (A) and in continuous flow (B) configurations**

**Two-Liter Scale**

***Copper-Carbon* welding rods as electrodes**

The initial pH was 8.74 ± 0.08. The average concentration of free chlorine was 630 ± 95 ppm. The turbidity varied more and was higher on average than the carbon-carbon setup, at 6.82 ± 3.58 NTU. The average current was 6.66 ± 0.07 Amperes. The connection may prove to be a very high benefit in ensuring the copper on the welding rod functions as the cathode. However, the consequence appears to be murkier and more suspended solids in the disinfectant.


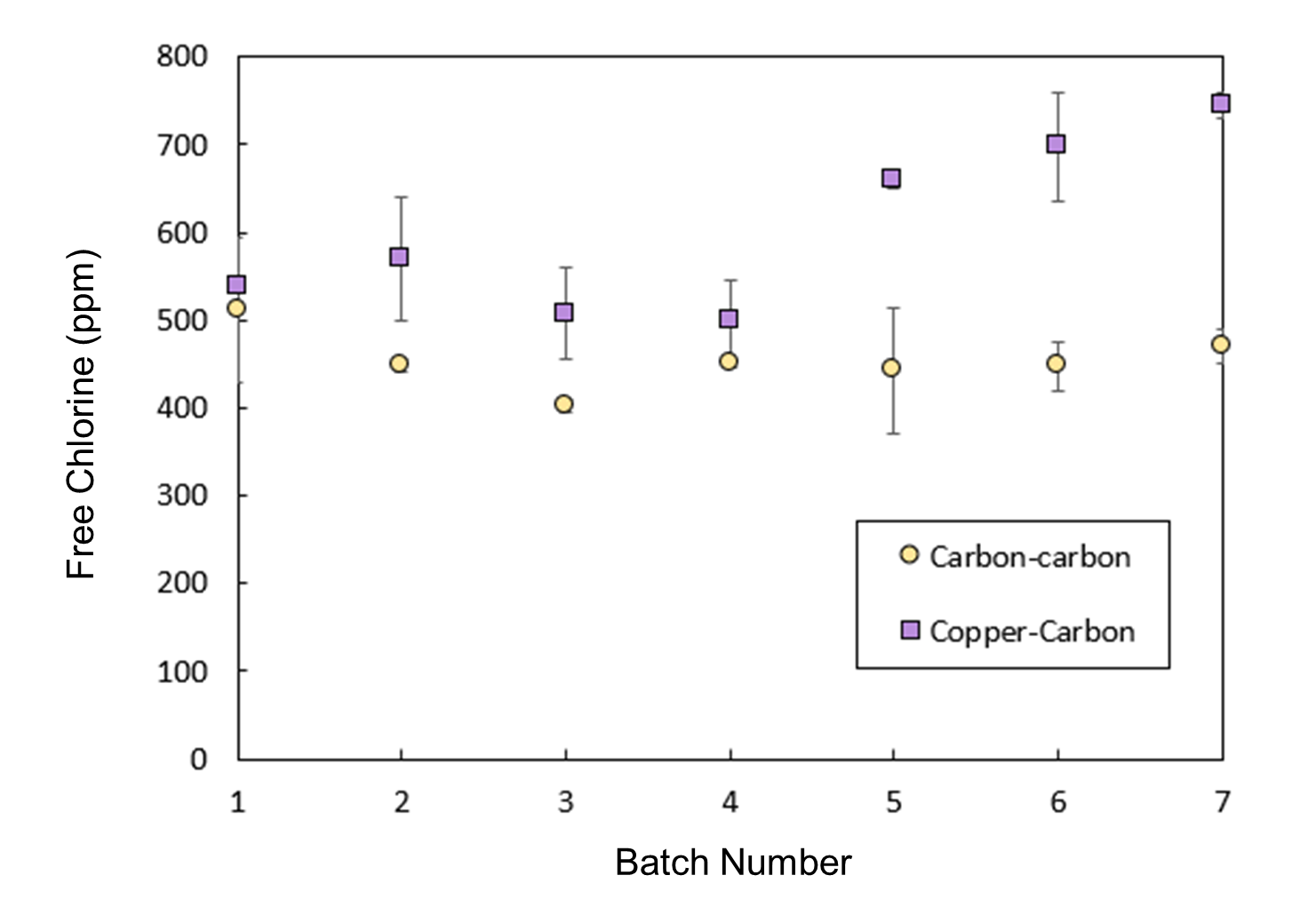


**Fig G. Comparison of free chlorine concentration over time of copper-coated carbon cathode (C-Cu) and bare carbon cathode.**

**Multi-Electrode Configurations**

In an attempt to increase free chlorine production in the 2-L scale Electro-Clean process, our team in Berkeley in partnership with our co-author Mr. Vijay Matange in India, experimented with various multi-electrode reactor configurations. The materials and methods used in these experiments were analogous to those described in the main text of the manuscript. The only difference is the number of electrodes used. There were three configurations included: 2 anode with 1 cathode, 1 anode with 2 cathode, and 1 anode with 1 cathode (as a control). Each configuration was operated in a separate 1.5-L plastic bottle with its own power supply. This procedure, of operating the three different reactor configurations simultaneously and in batch mode, was repeated three times to yield triplicate results for each of the reactor configurations. Error bars on Fig H (A, B, and C) indicate the standard deviation of the three trials.

As seen in Fig H (A, B, and C), a higher average current during electrolysis yields greater free chlorine production. However, Faradaic efficiency seems to drop slightly in configurations operating at a higher current. Since Faradaic efficiency of the electrode is not of great practical importance in the generation of a low-cost disinfectant, the use of additional electrodes during electrolysis could be advantageous as it does lead to increased free chlorine production during the same amount of time. However, one should also consider 1) how the electrodes may disintegrate faster over time when operating at a higher current, 2) how the cost of using additional electrodes compares to the benefit of generating slightly more free chlorine, and 3) testing additional configurations to increase electrode surface area and/or searching for carbon gouging rods of greater diameters that could also provide the additional surface area.


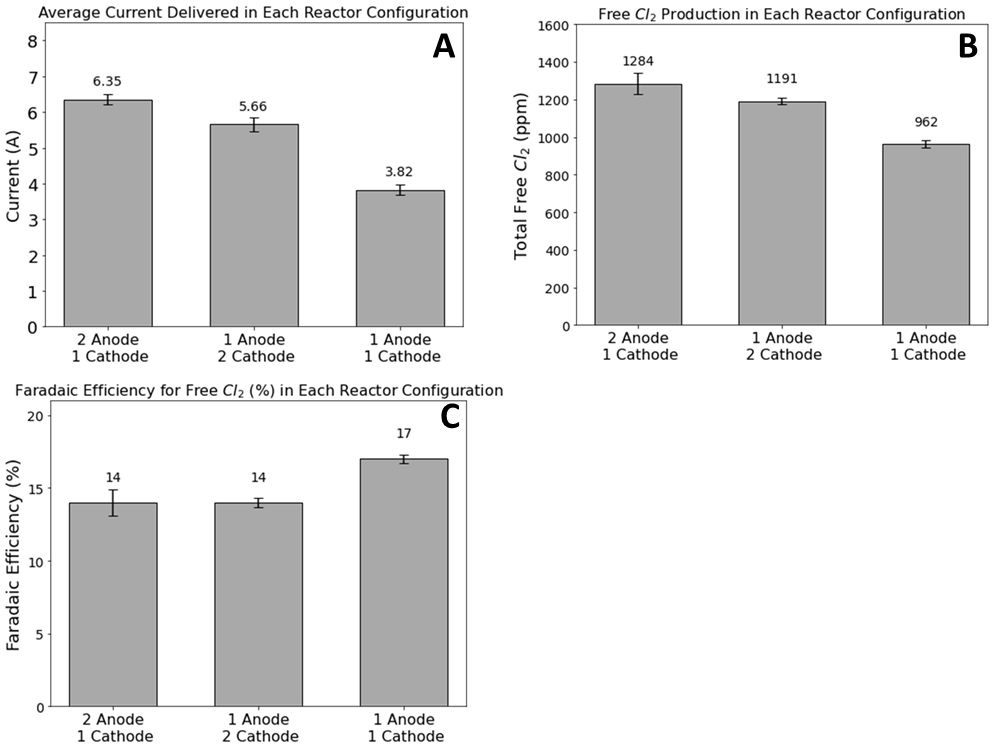


**Fig H.** **Performance of three electrode configurations.** (A) Average current delivered in each reactor configuration. (B) Free Cl_2_ production in each reactor configuration. (C) Faradaic efficiency for free Cl_2_ (%) in each reactor configuration.
