# Supplementary material for: Low-cost, local production of a safe and effective disinfectant for resource-constrained communities
Source: PLOS Glob Public Health. 2024 Jun 25;4(6):e0002213. doi: 10.1371/journal.pgph.0002213 (PMC11198905; doi:10.1371/journal.pgph.0002213)
Supplement: S4 Appendix — (DOCX) [file pgph.0002213.s004.docx]

**S4 Appendix. Long-term Experiments.**

A continuous-flow reactor assembly was used to test the durability of the carbon gouging anode. The continuous-flow system consisted of a feed tank with 30,000 mg/L table salt (Diamond Crystal brand) in tap water, a peristaltic pump with a flow rate of 0.25 mL/s, a 1.5-Liter plastic bottle with an inlet tubing at the bottom and an outlet tubing at the top, as shown in Fig A. The outlet tubing was set at a height that marked the 1.35-Liter volume.


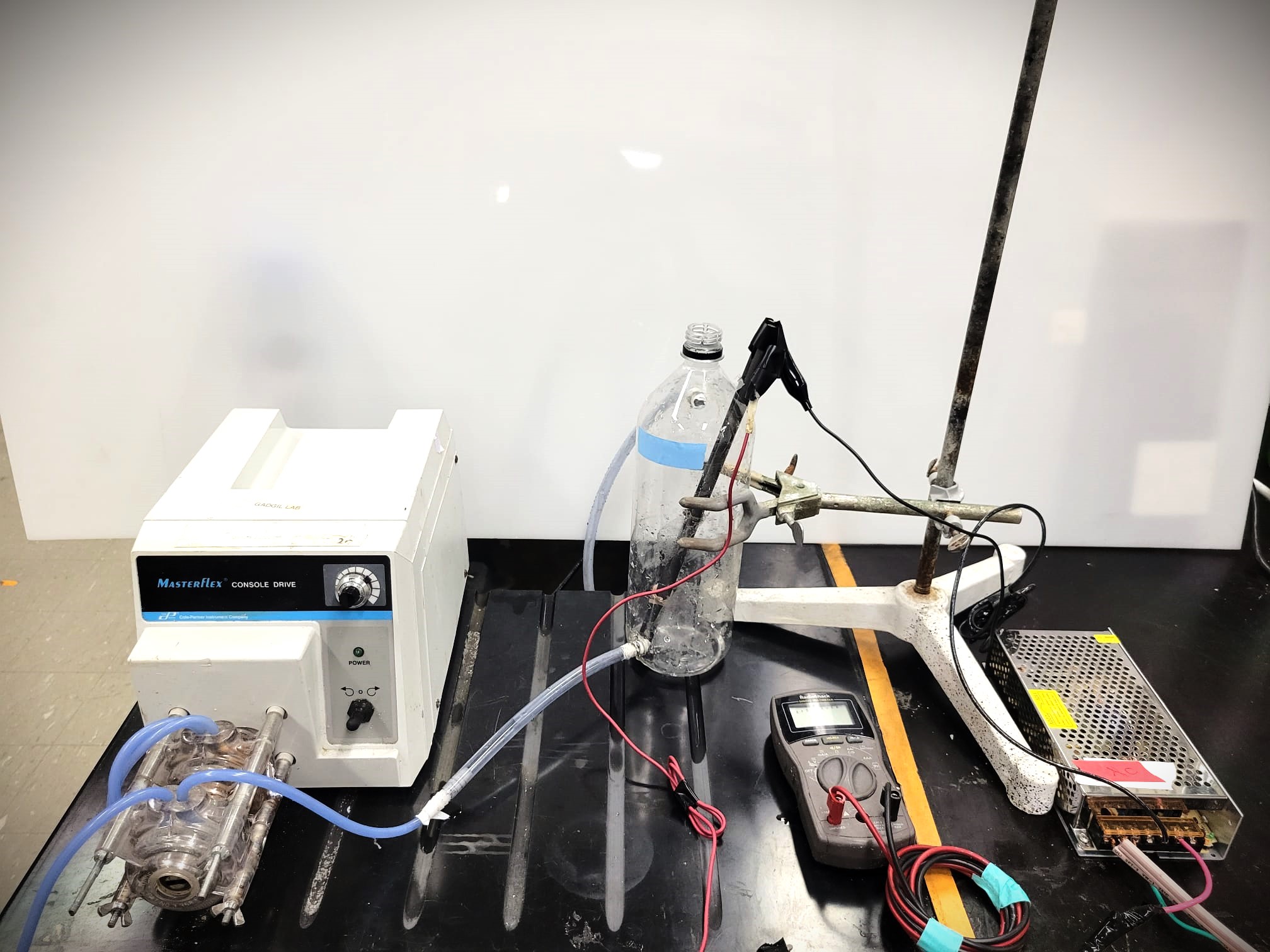


Peristaltic Pump

Inlet tubing

SMPS

Multimeter

Outlet tubing

Electro-Clean assembly

**Fig A. Assembly of continuous flow reactor system for testing the durability of the carbon gouging anode.**

**Durability of Mineral-Oil-Coated Electrodes**

The results of the long-term experiments using mineral-oil-coated gouging electrodes are shown below. Three trials were performed. In Figs S11 through S13, the horizontal dashed red line represents the average free chlorine concentration of all trials. The horizontal blue dashed lines represent the standard deviation, and the vertical dashed line represents the average charge passed through the electrodes.


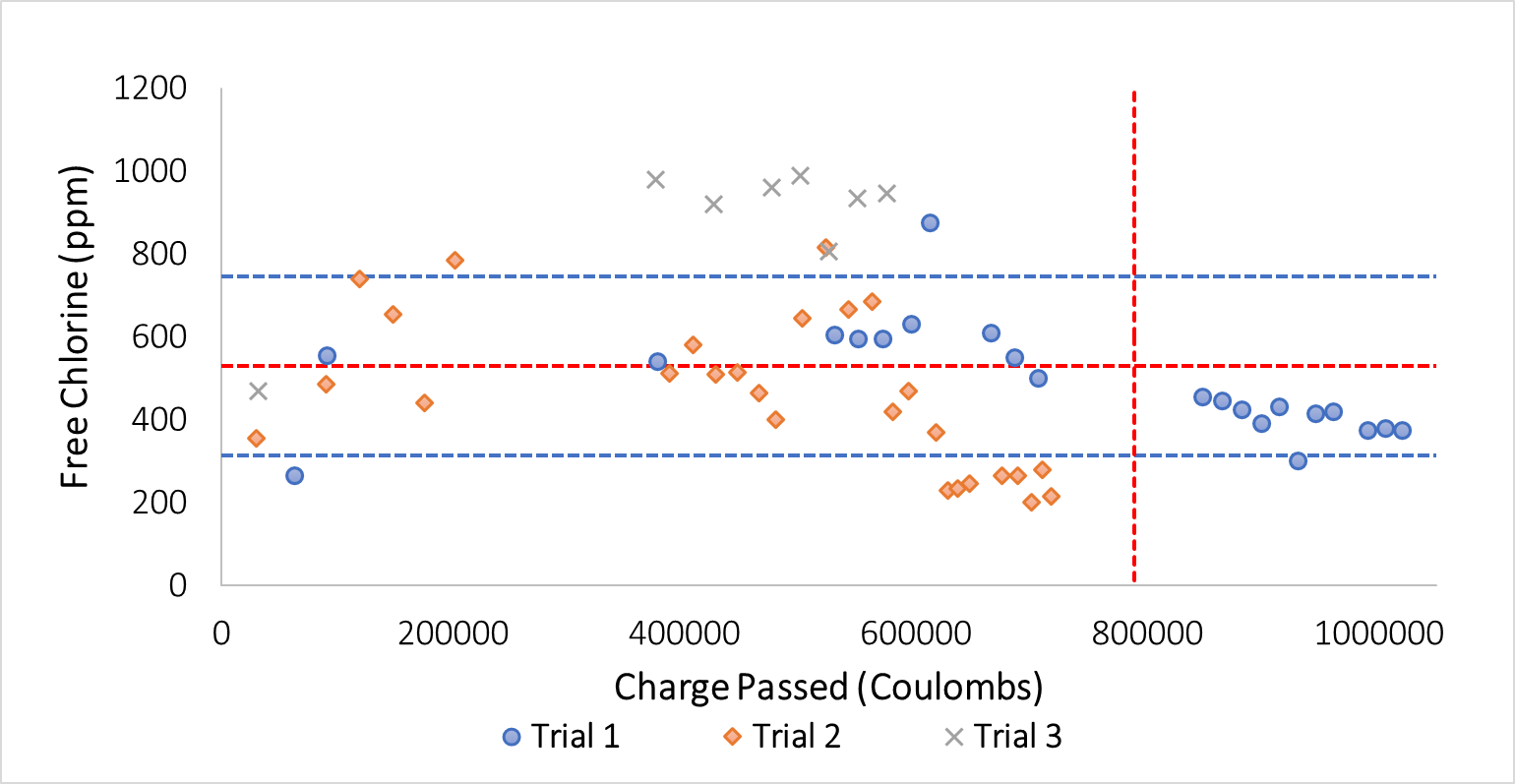
**Fig B. Free Chlorine concentration as a function of charge passed in Coulombs using mineral-oil-coated electrodes.**


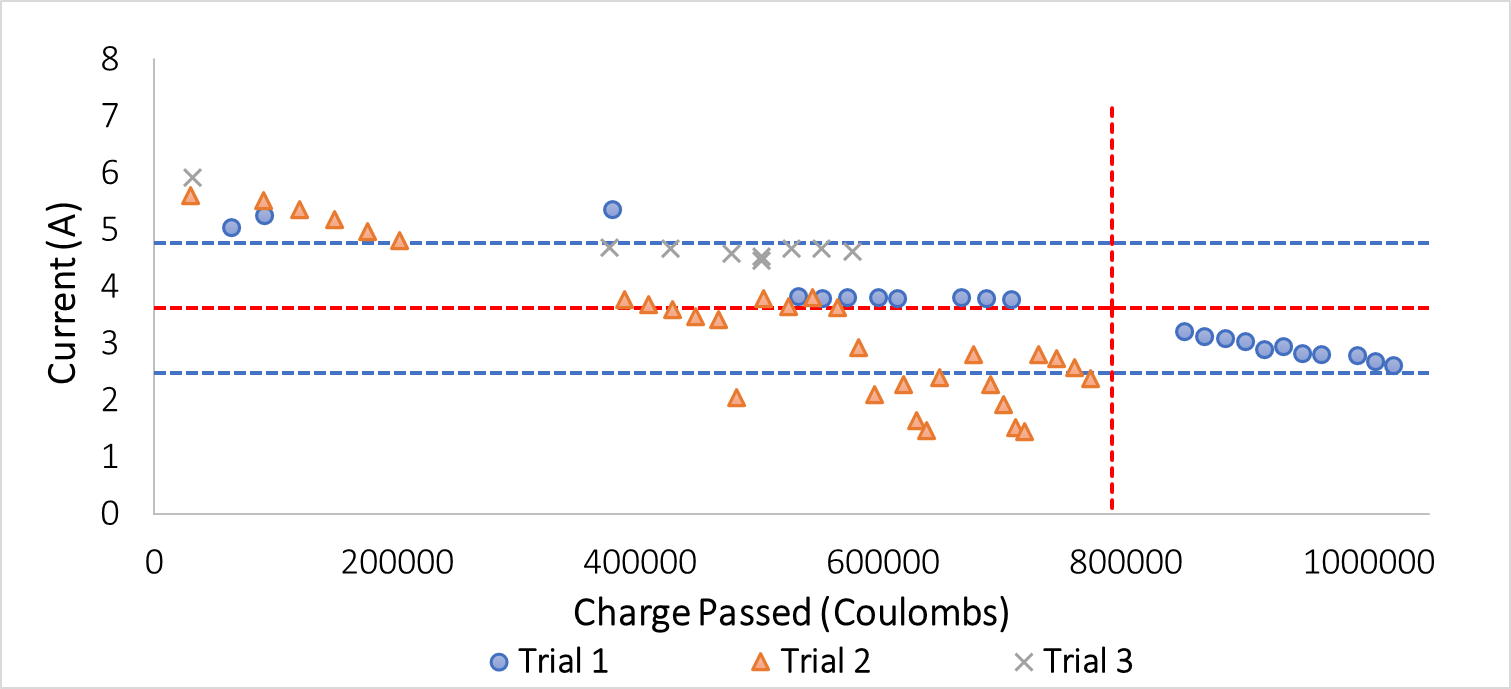
**Fig C. Current as a function of charge passed in Coulombs using mineral-oil-coated electrodes.**


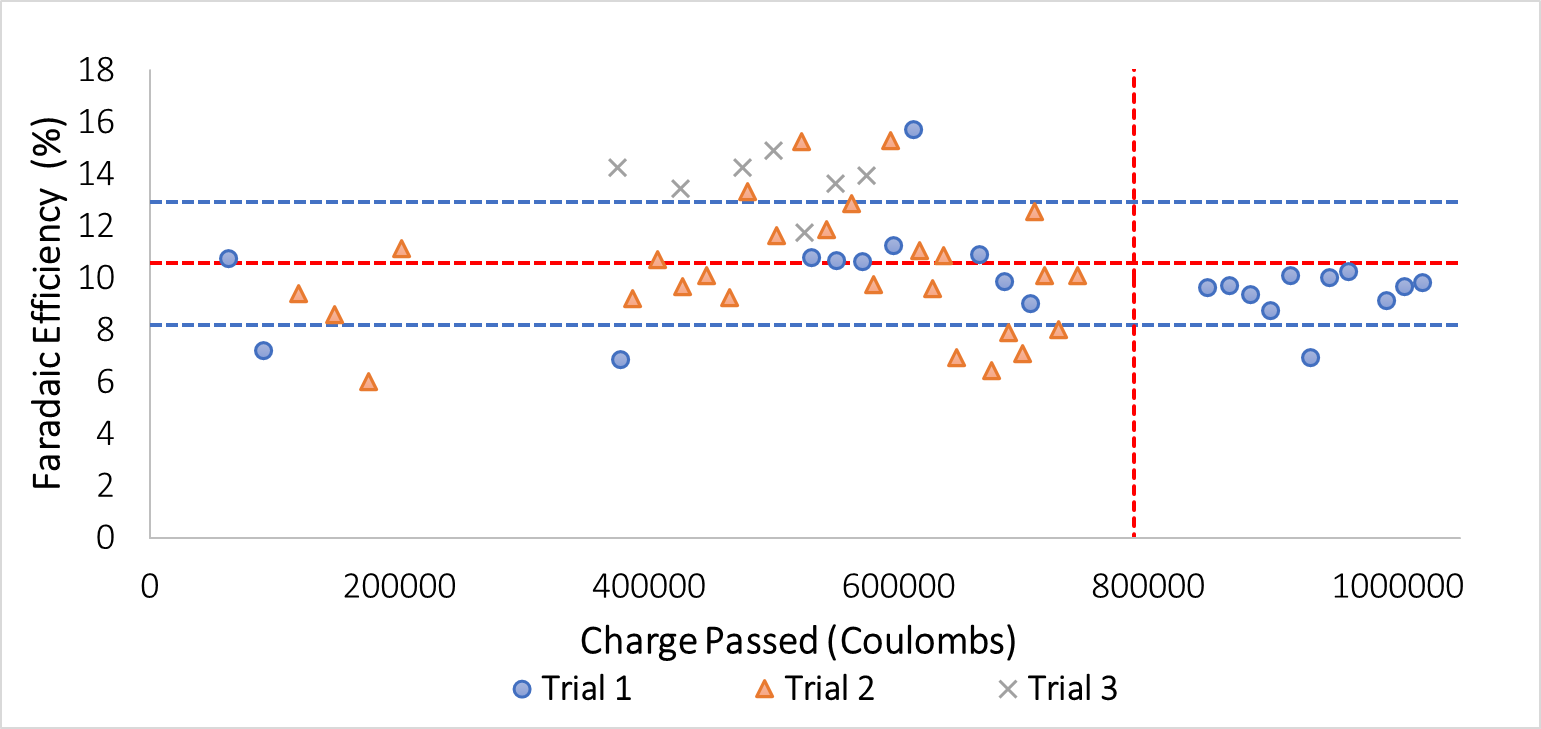


**Fig D. Faradaic efficiency as a function of charge passed in Coulombs using mineral-oil-coated electrodes.**

**Durability of Bare Carbon Electrodes**


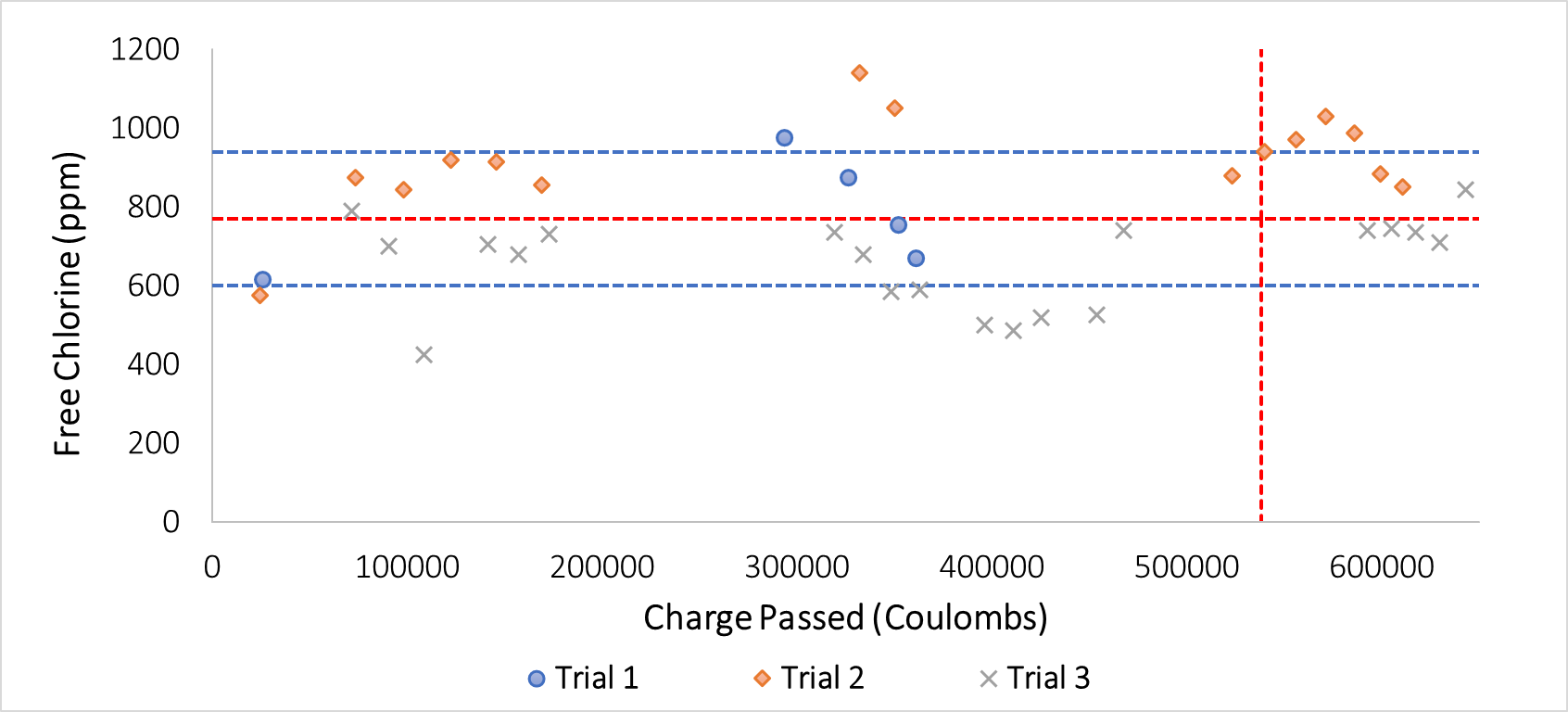
The results of the long-term experiments using bare carbon gouging electrodes are shown below. Three trials were performed. In Fig E through G, the horizontal dashed red line represents the average free chlorine concentration of all trials. The horizontal blue dashed lines represent the standard deviation, and the vertical dashed line represents the average charge passed through the electrodes.

**Fig E. Free Chlorine concentration as a function of charge passed in Coulombs using bare carbon electrodes.**


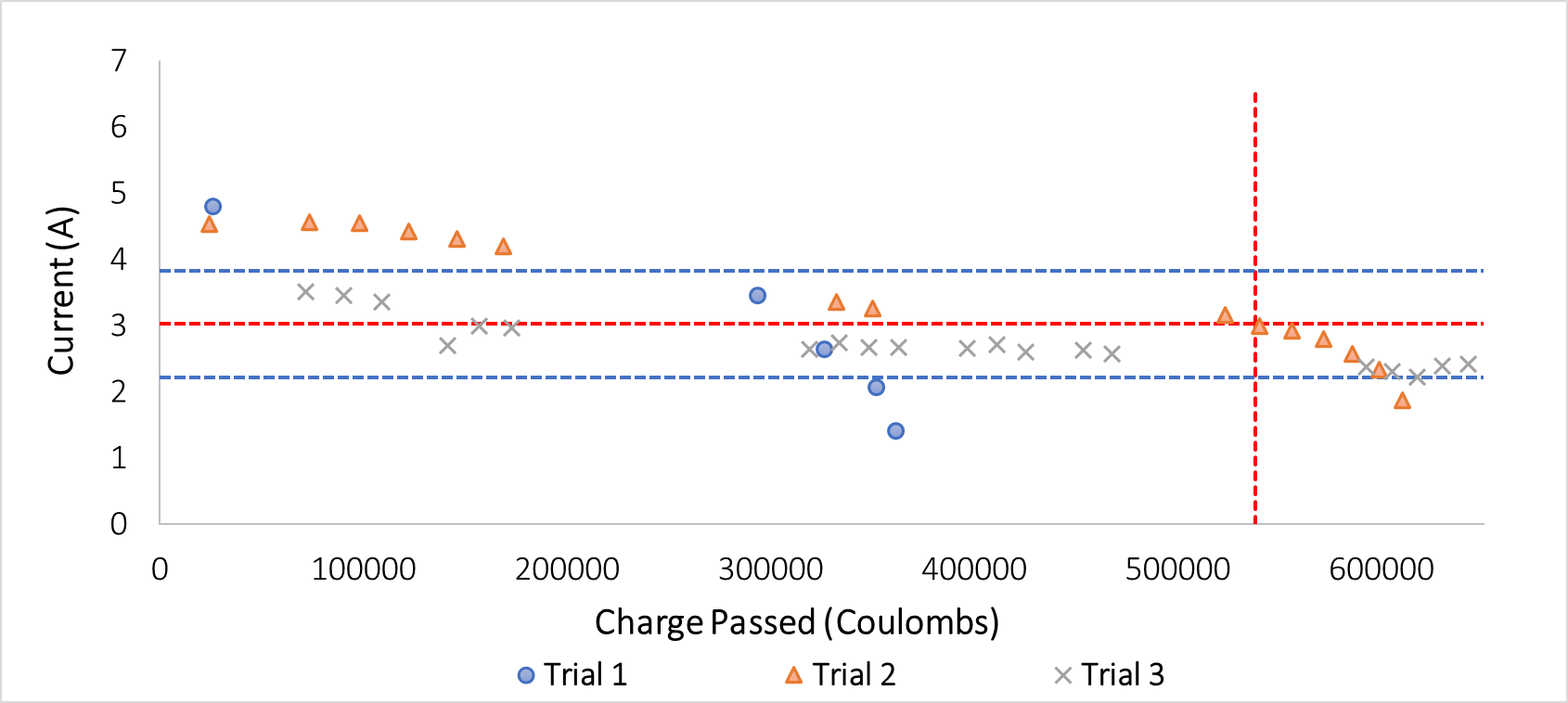
**Fig F. Current as a function of charge passed in Coulombs using bare carbon electrodes.**


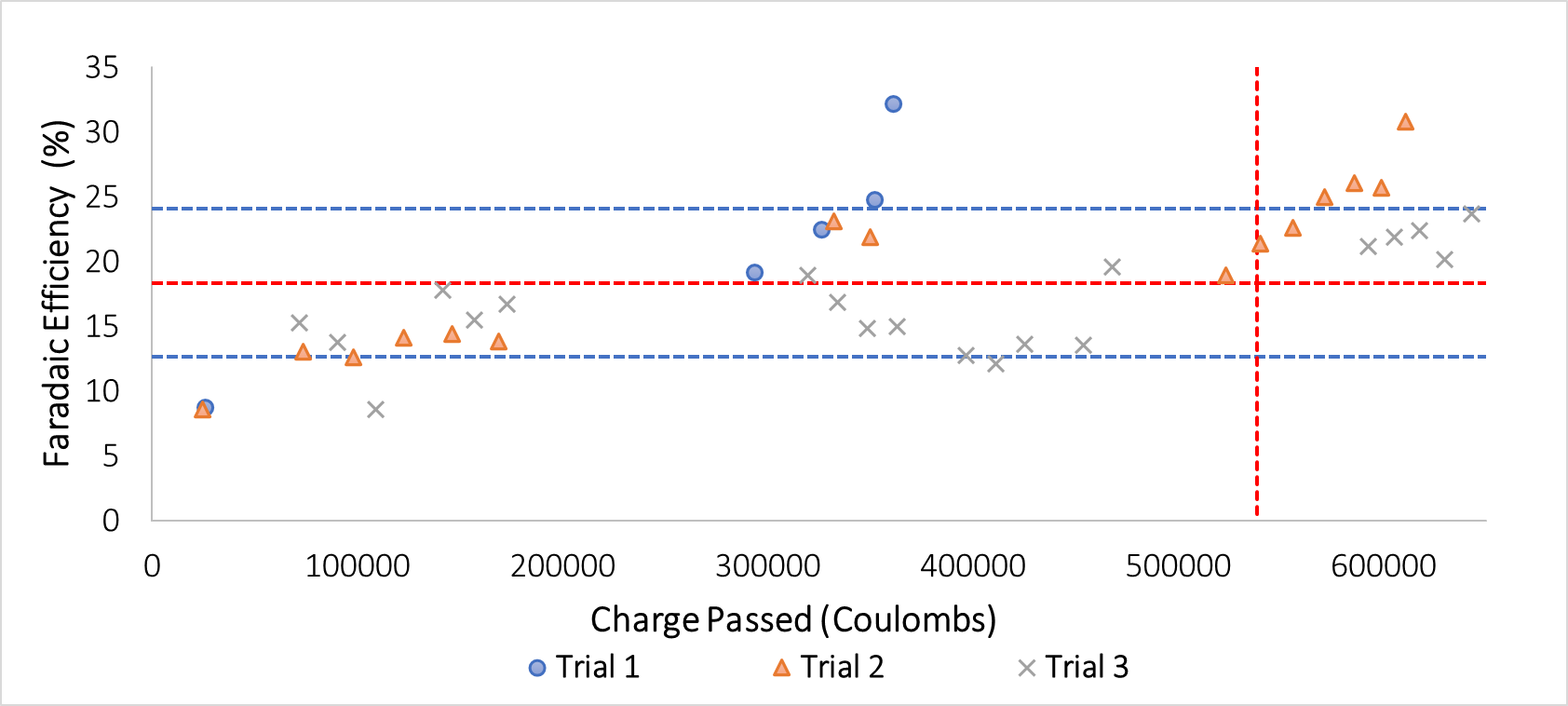
**Fig G. Faradaic efficiency as a function of charge passed in Coulombs using bare carbon electrodes.**

Variability within trials for mineral oil-coated and bare carbon gouging electrodes could be attributed to the corrosion of the alligator clips connecting the power supply and the electrodes. Over time, rust formed in the alligator clips, even when several windings of electrical tape were used to protect the alligator clips from moisture and some chlorine gas that escapes with the other gas bubbles. In practice, we recommend additional measures, such as adding anti-corrosion grease on any holes not protected by the electrical tape, to reduce the corrosion of the electrical connections to the electrodes.
