# Supplementary material for: Low-cost, local production of a safe and effective disinfectant for resource-constrained communities
Source: PLOS Glob Public Health. 2024 Jun 25;4(6):e0002213. doi: 10.1371/journal.pgph.0002213 (PMC11198905; doi:10.1371/journal.pgph.0002213)
Supplement: S5 Appendix — (DOCX) [file pgph.0002213.s005.docx]

**S5 Appendix. Microbiological Tests in Mexico****.**

***Microbial assays on various material surfaces***

This work was performed at the *Instituto Tecnológico Superior de Abasolo* (ITESA) in Mexico. Microbial strains were seeded on different material surfaces. Some surfaces were left as controls, others were sprayed on with a disinfectant. All were incubated and examined for bacterial growth. The evaluation of the bactericidal power of the HOCl solution produced by the Electro-Clean process in pure cultures was conducted using two bacterial strains, *Morganella morganii* and *Aerococcus viridans,* which are gram-negative and gram-positive, respectively. These strains were isolated from natural sources and identified by 16S rDNA sequencing. The strains were inoculated in 10 mL of Brain Heart infusion (BHI) broth and incubated at 30º C overnight for use in the assays. Luria-Bertani (LB) or BHI plates were used for strain maintenance.

We determined an initial concentration of 12.88*10^7^ CFU/mL for *A.viridans*, and 38.32*10^7^ CFU/mL for *M. morganii*. Prior to conducting the assays, the material surfaces were cleaned with tap water. We assayed three different surface materials (polystyrene, stainless steel, and glass) each with an area of 4 cm x 10 cm. We used 200 µL of each strain and distributed it with sterile cotton on the surface. For *A. viridans* the total CFU for the surface tested was 25.76*10^6^ CFU/200 µL and for *M. morganii* was 76.64*10^6^ CFU/200 µL. The surfaces were wiped with a wet cotton swab in one direction with a) Sterile water, b) Ethanol 70% (v/v), c) Bleach 1% (v/v), and d) produced 250 ppm as HOCl. As a blank, we only put the strains without treatment. The treated surfaces were allowed to dry before sampling with a sterile cotton swab and plated on a BHI agar plate. The plates were incubated at 30^o^C for 24 hours for colony development.

***Evaluation of antimicrobial effect of produced HOCl solution on various materials***

The bactericidal effect of the produced HOCl solution on various material surfaces is shown in Table A. The material type was observed to have an effect on microbial survival. The easiest material to clean was stainless steel, while the most difficult material to clean was polystyrene. Furthermore, the produced hypochlorous acid solution did not reduce CFU counts for the Gram-positive strain as effectively as it did for the Gram-negative strain. No one treatment was enough to completely eliminate the strains used in these assays. However, they all killed approximately 99.9% of the initial bacterial population. For most cases, except for *A. viridans* on polystyrene, the produced hypochlorous acid solution was able to reduce CFU counts below the suggested limit value of 200 CFU/40 cm^2^ for surfaces in public spaces [[1](https://www.zotero.org/google-docs/?yQNyux)]. To achieve cleaning standards for hospital surfaces (less than 100 CFU/ 40 cm^2^), higher concentrations would be suggested [[1](https://www.zotero.org/google-docs/?0wctN3)].

**Table A. Effect of Electro-Clean on different materials.** Values shown in this table have units of CFU per mL of strain solution on a 40 cm^2^ area sampled, with n=3. Percent removal is shown in parentheses for glass. Other materials showed ~99.9% removal.

| **Treatment** | **Glass** | | **Stainless steel** | | **Polystyrene** | |
| --- | --- | --- | --- | --- | --- | --- |
|  | ***A. viridans*** | ***M. morganii*** | ***A. viridans*** | ***M. morganii*** | ***A. viridans*** | ***M. morganii*** |
| **Blank** | Uncountable | Uncountable | Uncountable | Uncountable | Uncountable | Uncountable |
| **Water** | Uncountable | 1900 ± 775 (99.8%) | 1000 ±240 | Uncountable | Uncountable | 2390±550 |
| **Ethanol 70%** | 120 ±300  (99.9%) | 0  (100%) | 10 ±35 | 105 ± 125 | 5 ±5 | 290 ±200 |
| **Bleach 1%** | 170 ±90  (99.9%) | 60 ± 60  (99.9%) | 135 ±105 | 15 ± 20 | 80 ±50 | 985 ±985 |
| **Electro-clean 250 ppm** | 395 ±445  (99.9%) | 5 ±5  (99.9%) | 130 ±120 | 10 ±10 | 1490±980 | 140 ±190 |

The uncountable CFU in the plates indicates that the colonies were too high to count and were considered greater than 5000 CFU. This is a typical result for a blank sample without treatment and reflects the concentration as a result of applying 10^6^ CFU/mL in order to compare the effects of the disinfectant solution. The microbial CFU in all cases was reduced due to applying ethanol, bleach, and the produced hypochlorous acid solution. Some differences in CFU-reduction on various materials could be influenced by relevant material-surface properties (e.g., electrostatic charge) that have a bactericidal effect, for example, glass having a negative surface charge. The treatments reduce the bacterial population with an efficiency of 99.9% in most cases.

***Effect of produced HOCl solution on high-touch surfaces***

Two universities located in the cities of Irapuato and Abasolo in the state of Guanajuato Mexico were selected to analyze the effect of the hypochlorous acid solution produced by the Electro-Clean process on high-touch surfaces. Bathrooms and classrooms were selected as two areas to apply and verify the antimicrobial effect of the produced hypochlorous acid solution, considering these are the most visited and touched areas by students and teachers. For the evaluation of use in classrooms, the disinfecting solution was prepared to a final concentration of 300 ppm as HOCl just prior to use and was applied with a spraying device. The produced hypochlorous acid solution was applied on desks and tables, and the surfaces were sampled after 10 minutes of treatment on a surface area of approximately 7 cm^2^ with a sterile swab. The swab was then plated in either Luria-Bertani (LB) or Brain Heart Infusion (BHI) agar plates and incubated at room temperature for 24 hours until the development of the microbial colonies. To facilitate the growth of airborne microbes, cultures were added to the agar plates without prior sterilization using a spirit lamp or alcohol burner.

***Effect of produced HOCl solution on high-touch surfaces at schools***

| **Table B. Effect of produced HOCl used**  **in school classrooms.**   \| **Surface** \| **Classrooms (n=3)** \| \| \| --- \| --- \| --- \| \| Before Treatment (CFU / 7 cm^2^) \| After Treatment (CFU / 7 cm^2^) \| \| Desk \| 92 ± 67 \| 4 ± 7 \| \| Table \| 69 ± 49 \| 7 ± 3 \| | **Table C. Effect of produced HOCl used**  **in school bathrooms.**   \| **Surface** \| **Bathrooms (n=5)** \| \| \| --- \| --- \| --- \| \| Before Treatment (CFU / 7 cm^2^) \| After Treatment (CFU / 7 cm^2^) \| \| Toilet \| 223 ± 170 \| 13 ± 14 \| \| Door \| 43 ± 40 \| 15 ± 11 \| \| Sink \| 48 ± 21 \| 5 ± 5 \| |
| --- | --- | --- | --- | --- | --- | --- | --- | --- | --- | --- | --- | --- | --- | --- | --- | --- | --- | --- | --- | --- | --- | --- | --- | --- | --- | --- |

The results of applying the hypochlorous acid solution produced through the Electro-Clean process (diluted to 250 ppm as free chlorine, pH 6) in classrooms and bathrooms are shown in Tables B and C, respectively. The CFU count of the microbial population dropped but was not completely eliminated. This result suggests that a higher concentration of HOCl is needed for surface disinfection in public spaces, especially high-touched surfaces in bathrooms such as toilets and doorknobs to achieve even lower CFU counts.

***References***

[1. Querido MM, Aguiar L, Neves P, Pereira CC, Teixeira JP. Self-disinfecting surfaces and infection control. Colloids Surf B Biointerfaces. 2019 Jun 1;178:8–21.](https://www.zotero.org/google-docs/?T9AxrV)
