# Supplementary material for: Low-cost, local production of a safe and effective disinfectant for resource-constrained communities
Source: PLOS Glob Public Health. 2024 Jun 25;4(6):e0002213. doi: 10.1371/journal.pgph.0002213 (PMC11198905; doi:10.1371/journal.pgph.0002213)
Supplement: S6 Appendix — (DOCX) [file pgph.0002213.s006.docx]

**S6 Appendix. Microbiological Tests in India.**

Microbiological surface testing methods aligned with those of international standards [[1–](https://www.zotero.org/google-docs/?HvwrfR)4].

***Pathogen removal efficiency of produced HOCl solution***

Gram-negative intestinal enteric bacteria (such as *Escherichia coli, Enterobacter, Klebsiella, Salmonella, Proteus species, Yersinia, Pseudomonas aeruginosa,* and *Shigella*) were used in this study to test the efficacy of the produced hypochlorous acid solution in disinfecting pathogens. Colony-forming units (CFU) assays were conducted as per protocol APHA-AWWA-WEF 9215 B with MacConkey agar. This agar is a selective medium with crystal violet and bile salts that inhibit the growth of gram-positive organisms and allow the growth of Gram-negative organisms. The agar plates were closed to avoid culture contamination but not hermetically sealed to allow contact with atmospheric oxygen. At the end of the incubation period, the number of colonies in each plate was determined using OpenCFU software (3.8 Beta, open access). The efficiency of the produced hypochlorous acid solution to stop the spread of pathogens was verified as (i) a surface disinfectant and (ii) a growth inhibitor.

***Growth inhibitor evaluation***

Known amounts of pathogenic bacterial cultures were pipetted on sterilized Petri plates containing hardened agar (spread-plated) and were allowed to sit for 5 minutes. Subsequently, half of the area of the Petri plates was treated with 100 µL of one of the following three different solutions: water, ethanol 70% (v/v), or hypochlorous acid (300 ppm, pH 6). Plating was performed in duplicates for each of the solutions and incubated for 24 hours, at 30º C.

***Hypochlorous acid as a growth inhibitor for pathogenic bacterial strains***

Hypochlorous acid and ethanol have been used as growth inhibitors for the growth of pathogenic bacteria. As shown in Fig A(A), hypochlorous acid is efficient in reducing the growth of the group of non-lactose fermenting pathogenic bacteria (i.e., *Salmonella, Proteus species, Yersinia, Pseudomonas aeruginosa, and Shigella*) from 2930 to 103 CFU (a reduction of 10^-1^), whereas inefficient in reducing the growth of lactose fermenting pathogenic bacteria (i.e.,*Escherichia coli, Enterobacter,* and *Klebsiella*) as shown in Fig A(D). Ethanol could reduce non-lactose fermenting pathogen bacteria from 2390 to 987 CFU as shown in Fig A(B) and lactose fermenting pathogen bacteria from 7500 to 32 CFU as shown in Fig A(E). Distilled water could not reduce the growth of pathogen bacteria, as shown in Fig A(C) and Fig A(F).

| **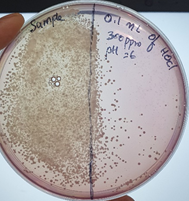**  **(A)** | **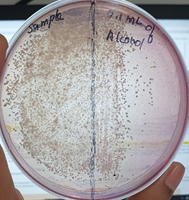**  **(B)** | **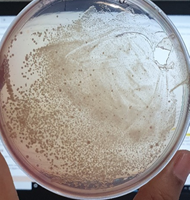**  **(C)** |
| --- | --- | --- |
| **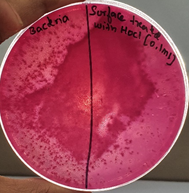**  **(D)** | **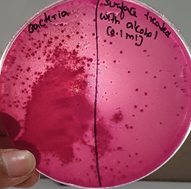**  **(E)** | **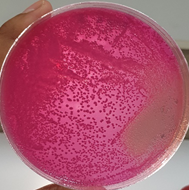**  **(F)** |

**Fig A. Digital pictures of pathogenic bacterial growth inhibition by hypochlorous acid and ethanol.** Growth inhibition of non-lactose fermenting pathogen bacteria (*Salmonella, Proteus species, Yersinia, Pseudomonas aeruginosa*, and *Shigella*) by (A) hypochlorous acid solution, (B) Ethanol 70% (v/v), and (C) Water. Growth inhibition of lactose fermenting pathogenic bacteria: *Escherichia coli, Enterobacter*, and *Klebsiella* by *(*D) hypochlorous acid solution, (E) Ethanol 70% (v/v), and (F) Water.

***Evaluation of surface disinfection of pathogenic cultures***

A known quantity of pathogenic bacterial cultures (i.e., 900 × 10² to 3900 × 10² *Escherichia coli* CFU/mL and 112 × 10⁴ to 210 × 10⁵ *Salmonella enterica* serotype Choleraesuis CFU/mL) was spread onto various materials (glass, aluminum, plastic) and dried. The surfaces were then treated with 100 µL of either 70% ethanol (v/v) or 100 µL of hypochlorous acid (300 ppm, pH 6) and allowed to dry for 5 minutes. The treated surfaces and controls were subsequently placed in sterilized Petri dishes containing solidified agar and incubated for 24 hours at 30°C.


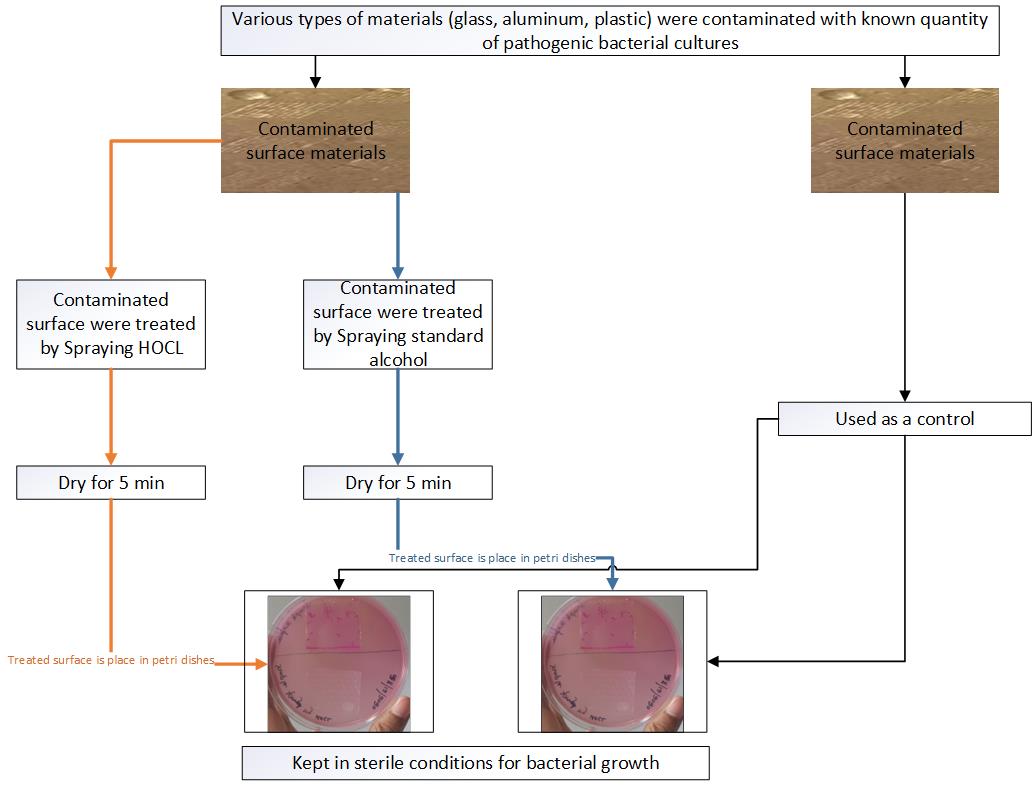


**Fig B.** **Process flow diagram for the methodology of evaluation of surface disinfection of pathogenic cultures.**

***Hypochlorous acid as a surface disinfectant on various material surfaces***

The surface was artificially contaminated with pathogenic bacteria, specifically *Escherichia coli* an*d Salmonella enterica* serotype *Choleraesuis*, with bacterial loads ranging from 900x10² to 3900x10² CFU/mL and 112x10⁴ to 210x10⁵ CFU/mL, respectively. The contaminated surface was then treated with a combination of hypochlorous acid solution and ethanol, following the methodology described in *Evaluation of surface disinfection of pathogenic cultures*. As shown in Fig C (A, B, and C), the produced hypochlorous acid (300 ppm free-chlorine, pH 6) was modestly effective in disinfecting the contaminated aluminum surface, whereas for plastic and glass surfaces, it could reduce the growth of only *Escherichia coli*. Ethanol was effective in disinfecting all surfaces, as shown in Fig C (D, E, and F).

| 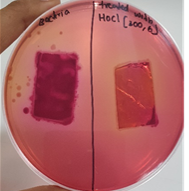 | 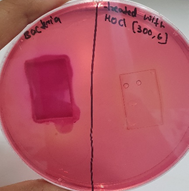 | 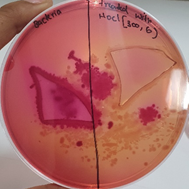 |
| --- | --- | --- |
| **(A)** Aluminum surface treated with hypochlorous acid | **(B)** Plastic surface treated with hypochlorous acid | **(C)** Glass surface treated with hypochlorous acid |
| 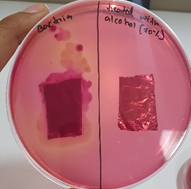 | 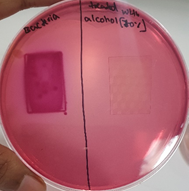 | 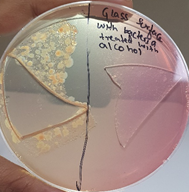 |
| **(D)** Aluminum surface treated with ethanol 70% v/v | **(E)** Plastic surface treated with ethanol 70% v/v | **(F)** Glass surface treated with ethanol 70% v/v |

**Fig C. Bacterial growth of *Escherichia coli* and *Salmonella enterica* serotype *Choleraesuis* on untreated and treated surfaces with 100 uL of HOCl (A-C), and ethanol 70% v/v (D-F).**

**References**

1. Hamilton MA. KSA-SM-05— Testing surface disinfectants: how the differences between disinfectant tests and chemical assays affect method evaluation criteria [Internet]. Bozeman, MT: Center for Biofilm Engineering at Montana State University; 2010 Aug. Available from: https://biofilm.montana.edu/documents/KSA-SM-05.pdf

2. Prince J, Ayliffe GAJ. In-use testing of disinfectants in hospitals. J Clin Pathol. 1972 Jul;25(7):586–9.

3. Stedman RL, Kravitz E, Bell H. Studies on the efficiencies of disinfectants for use on inanimate objects. I. Relative activities on a stainless steel surface using a new performance test method. Appl Microbiol. 1954 May;2(3):119–24.

4. Tamási G. Testing disinfectants for efficacy. Rev Sci Tech Int Off Epizoot. 1995 Mar;14(1):75–9.
