# Supplementary material for: Low-cost, local production of a safe and effective disinfectant for resource-constrained communities
Source: PLOS Glob Public Health. 2024 Jun 25;4(6):e0002213. doi: 10.1371/journal.pgph.0002213 (PMC11198905; doi:10.1371/journal.pgph.0002213)
Supplement: S7 Appendix — (DOCX) [file pgph.0002213.s007.docx]

**S7 Appendix. Scale-up Efforts by Co-Authors.**

As of January 2023, the Electro-Clean project has 15 partners in 10 countries around the world. Contributions and expertise of project collaborators varied, each bringing unique insights and additions to the project as a whole. This section showcases some of the larger scale-up efforts by our longtime collaborators in Nigeria, Mexico, and India.

***Covenant University in Ota, Nigeria***

Photos courtesy of co-author Professor David Omole and his students.

**
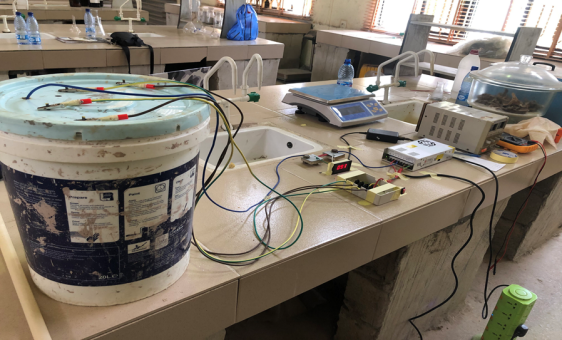
**

**(A)**

**
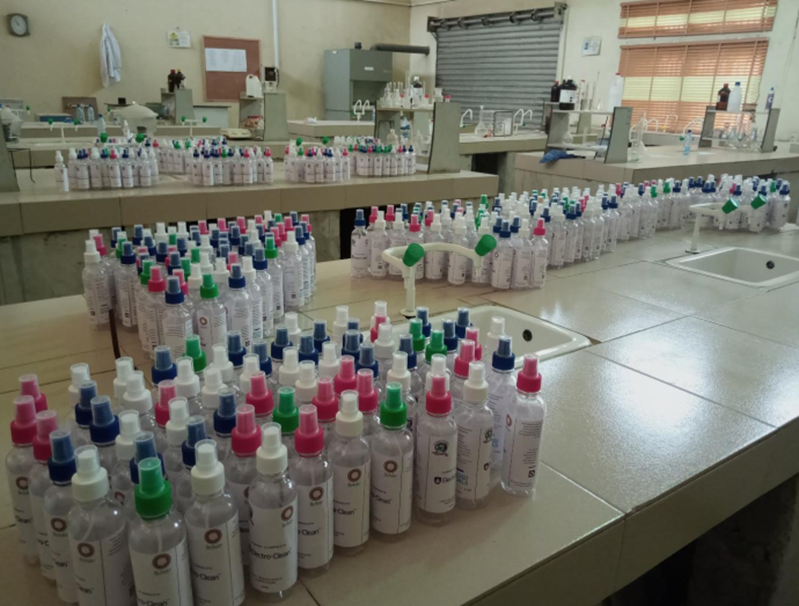

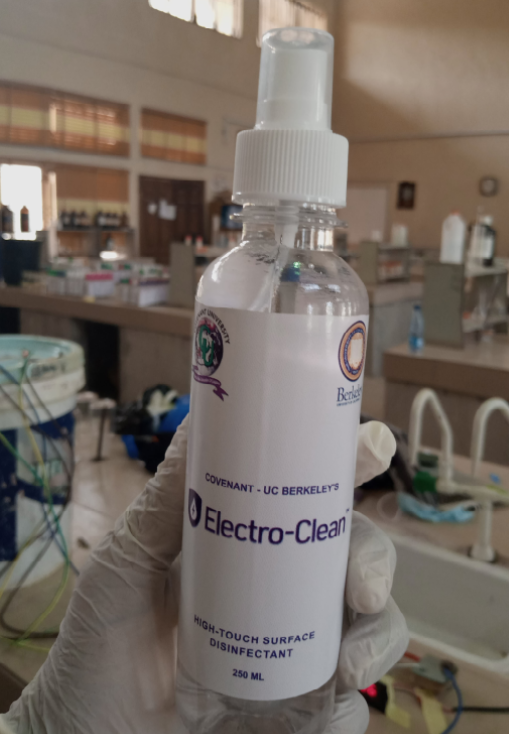
**

**(B) (C)**

**Fig A. Digital images of Electro-Clean process developed in Nigeria.** (A) Experimental set-up for Electro-Clean production at Covenant University, (B) More than 500 labeled spray bottles of the ready-to-use disinfectant prepared for mass distribution, and (C) Spray bottle labels included brief user instructions, statement of regulatory approval by University authorities, and information on where the disinfectant solution could be refilled.

***ITS, Abasolo & ITS, Irapuato in Mexico***

Using locally available materials, co-authors in Mexico designed the Electro-Clean assembly using multiple electrodes to achieve higher free chlorine concentrations. From user feedback, it was learned that having excessive electrical components exposed made the system overwhelming. As a result, the SMPS as well as the digital multimeter were placed inside an electrical box, as shown in Fig B (B) and (C). To spray larger surface areas in schools, our collaborators in Mexico placed 100 ppb of the solution on a medium-sized atomizer spray bottle, shown in Fig B (D). Photos are courtesy of co-authors Cesar Alvarez-Mejia and Varinia Lopez-Ramirez.


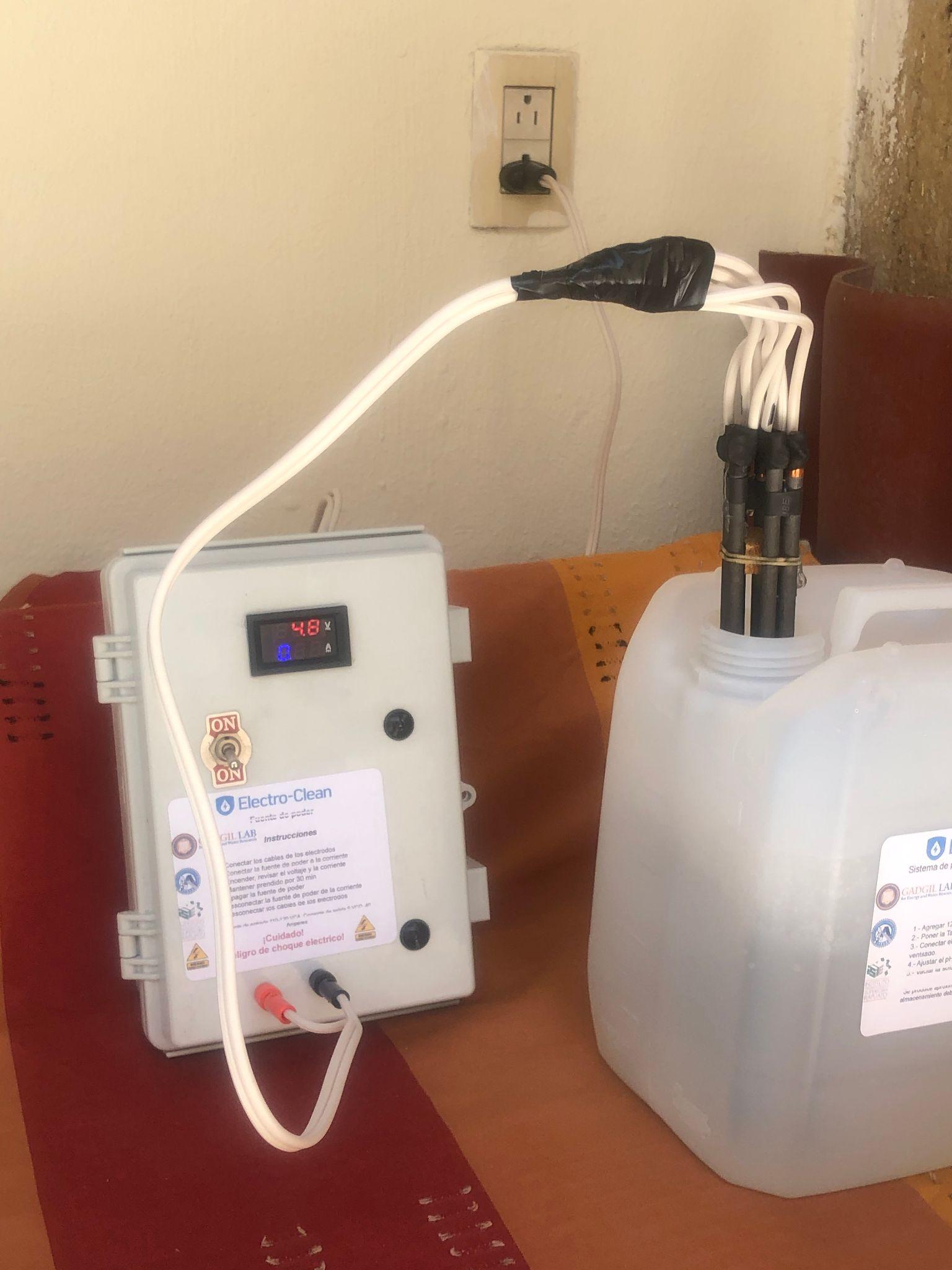

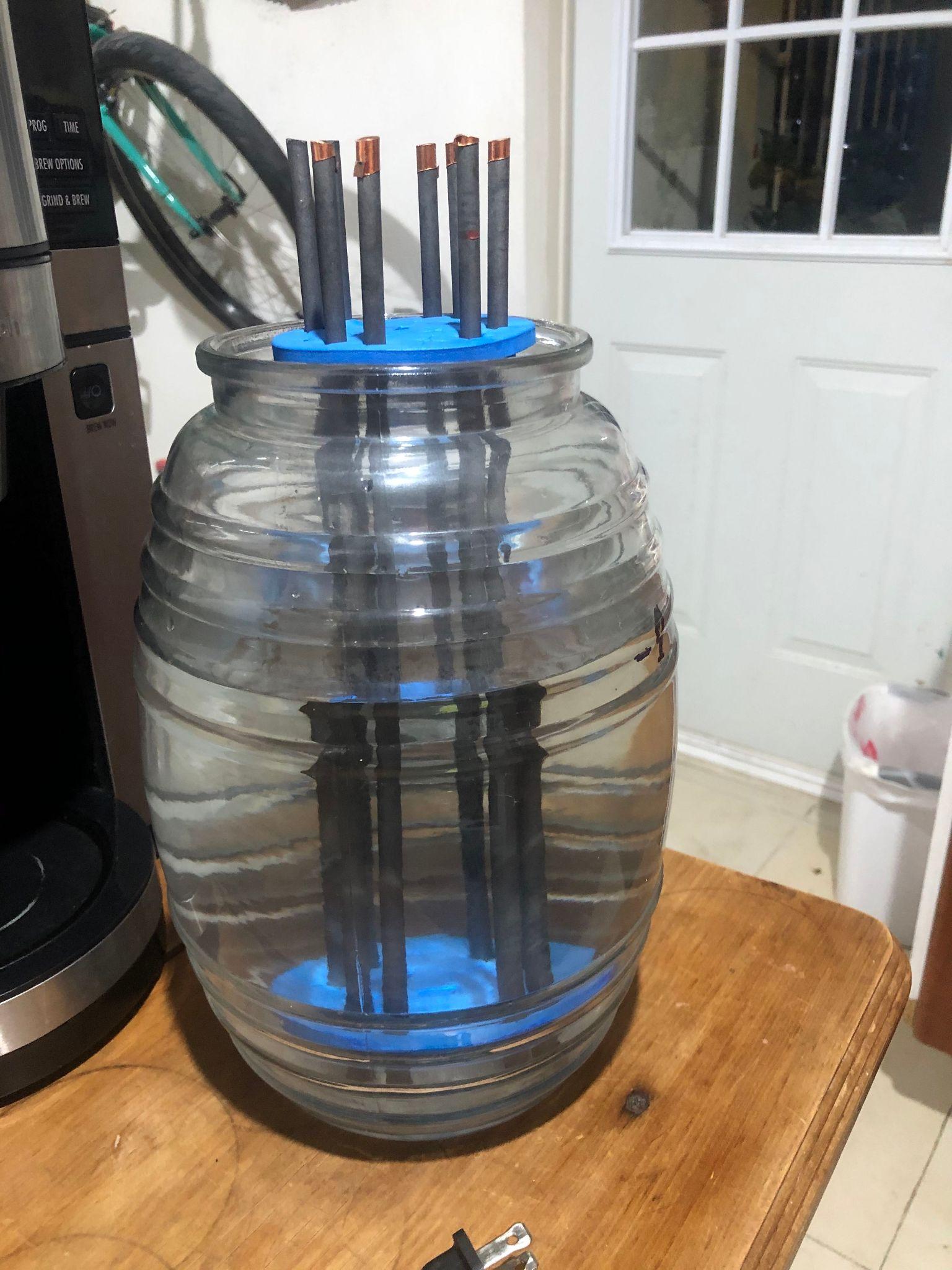


**A**

**B**


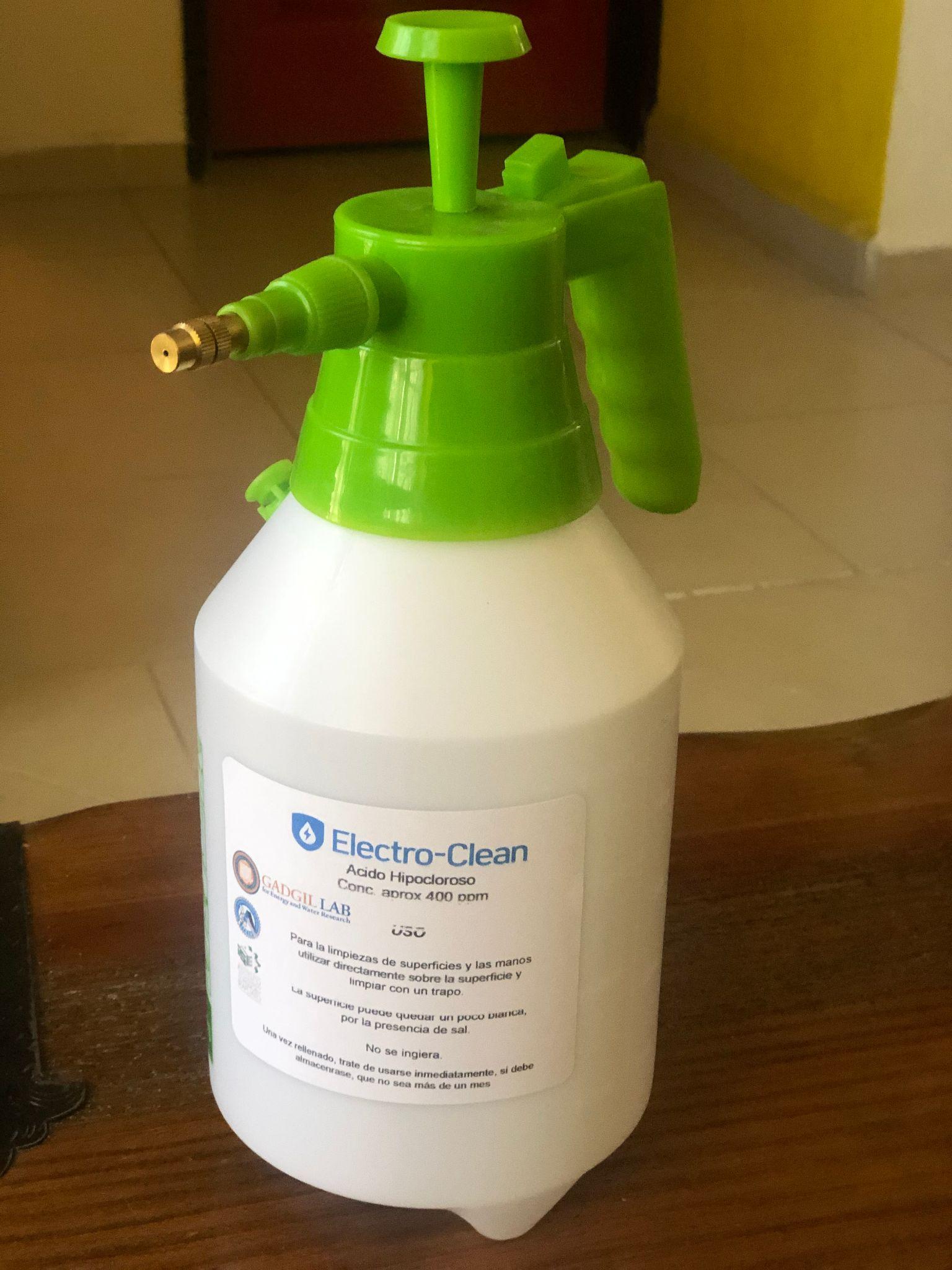

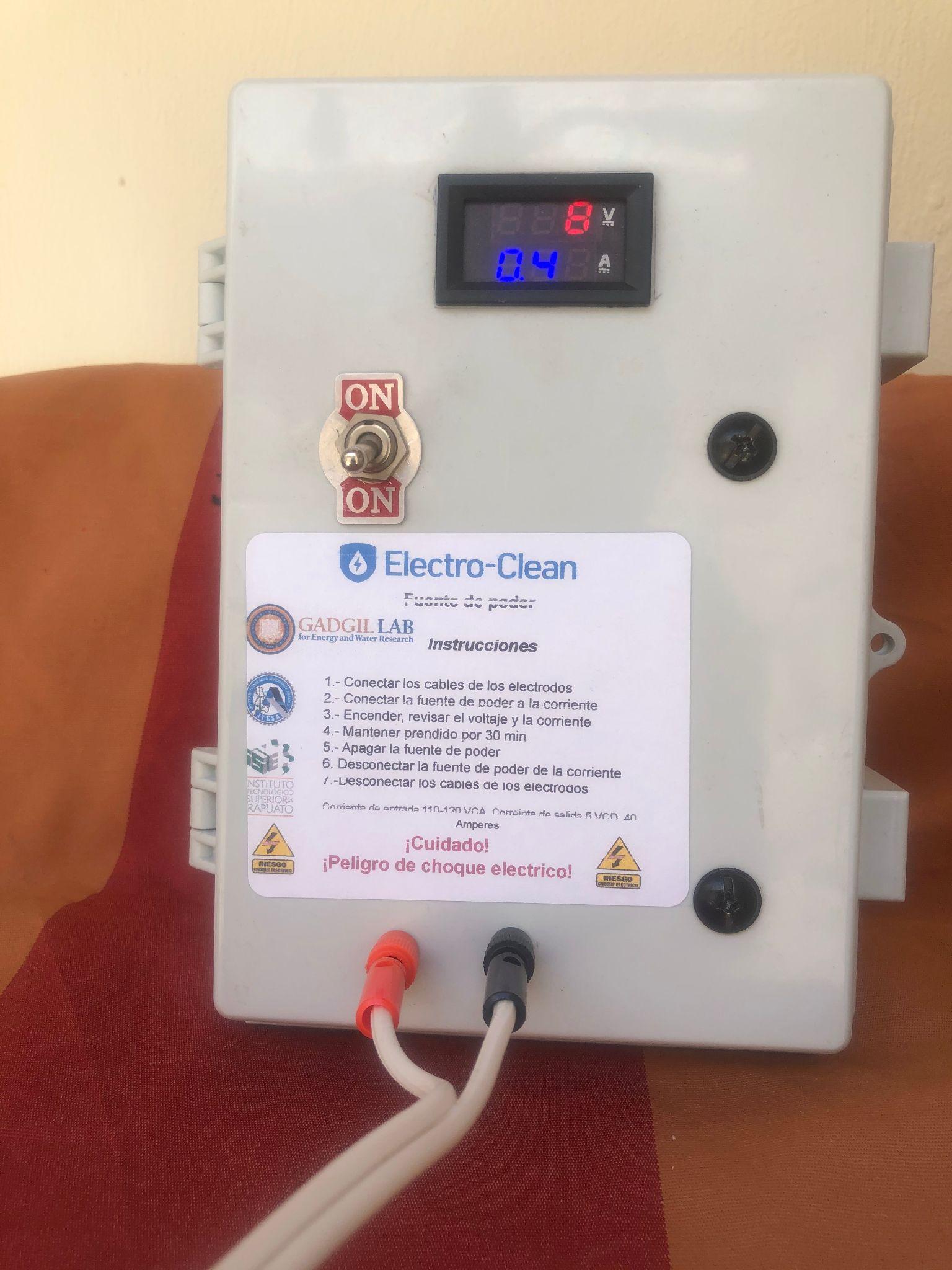


**D**

**C**

**Fig B. Digital images of Electro-Clean process developed in Mexico.** Digital images of (A) a large-scale reactor assembly with multiple electrodes, (B) a large-scale reactor assembly with all electrical components in an electrical box, (C) the electrical box with the electrical components inside, (D) the atomizer spray bottle used to disinfect larger areas.

**Vijay Matange of Vinyas Architects in India**

One of our co-authors in India, Mr. Vijay Matange, has been using the Electro-Clean production process since early 2020 as the disinfectant of choice for surface disinfection at the architectural consulting firm he owns and operates. Mr. Matange produces the concentrated solution on a regular basis and dilutes it at the time of use for disinfection of high-touch surfaces in the workplace and his home. He also conducted a wide range of experiments to increase the current delivered within the Electro-Clean system to increase free chlorine production, via improved electrical connections and multi-electrode configurations. Some selected photos are shown here. All photos are courtesy of Mr. Vijay Matange.

**
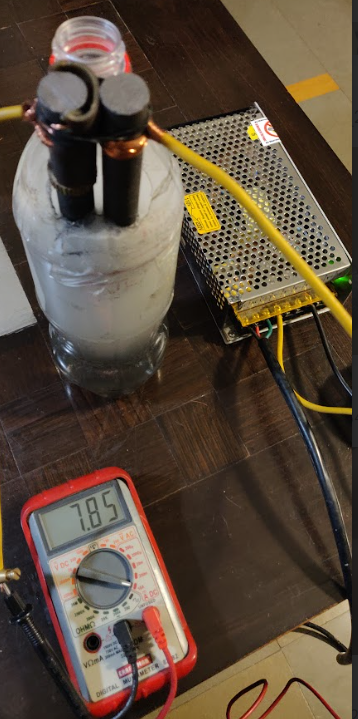

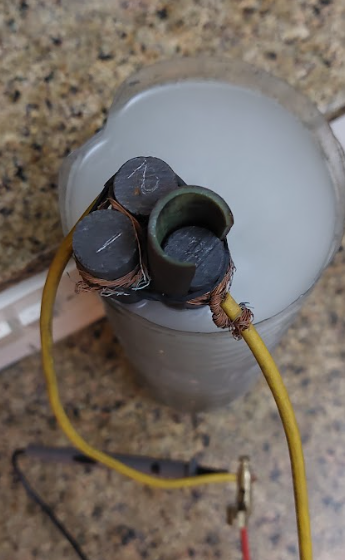

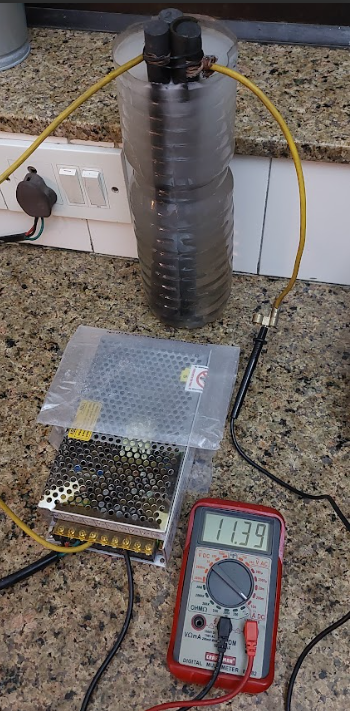
**

**(A) (B) (C)**

**Fig C. Digital images of Electro-Clean process developed in India**. (A) Standard Electro-Clean system at Vinyas Architects, (B) Dual-anode electrode configuration, and (C) Elevated current observed as a result of the dual-anode electrode configuration.

***Takataka Plastics in Gulu, Uganda***

Photos are courtesy of co-author Paige Balcolm from Takataka Plastics.


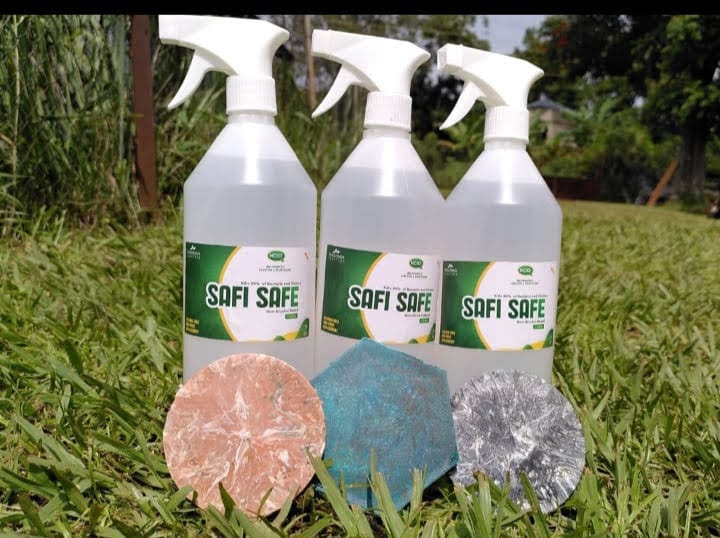


**(A)**


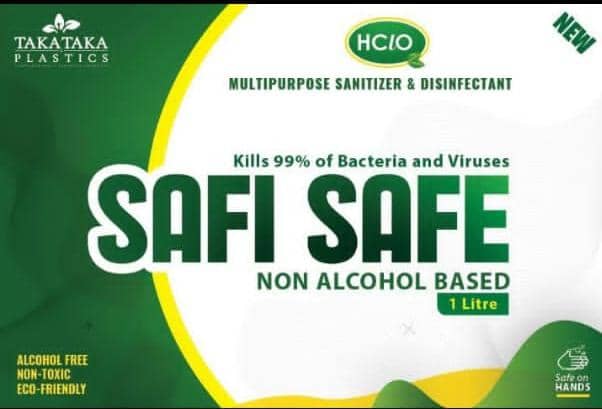


**(B)**

**Fig D. Digital images of Electro-Clean disinfectant made in Uganda**. The surface disinfectant was locally rebranded with the name “Safi Safe”. (A) Spray bottles of the surface disinfectant solution pictured with plastic tiles manufactured by Takataka plastic, and (B) close-up image of surface disinfectant label.
