# Supplementary material for: Low-cost, local production of a safe and effective disinfectant for resource-constrained communities
Source: PLOS Glob Public Health. 2024 Jun 25;4(6):e0002213. doi: 10.1371/journal.pgph.0002213 (PMC11198905; doi:10.1371/journal.pgph.0002213)
Supplement: S1 Table — Water Quality Data from EBMUD, Orinda Water Treatment Plant (accessed 06 February 2023). (DOCX) [file pgph.0002213.s008.docx]

**S1 Table. Electrolyte Composition.** Water Quality Data from EBMUD, Orinda Water Treatment Plant (accessed 06 February 2023).

| **Parameter** | **Unit** | **Value** |
| --- | --- | --- |
| Chloramine | mg/L | 2.69 |
| Fluoride | mg/L | 0.77 |
| Hardness | mg/L | 16 |
| pH | unitless | 9.4 |
| Temperature | ºC | 10.48 |
