# Supplementary material for: Low-cost, local production of a safe and effective disinfectant for resource-constrained communities
Source: PLOS Glob Public Health. 2024 Jun 25;4(6):e0002213. doi: 10.1371/journal.pgph.0002213 (PMC11198905; doi:10.1371/journal.pgph.0002213)
Supplement: S2 Table — (DOCX) [file pgph.0002213.s009.docx]

**S2 Table. Specifications of the locally available materials used by co-authors in India, Mexico, Nigeria, and Uganda.**

| **Material** | **India** | **Mexico** | **Nigeria** | **Uganda** |
| --- | --- | --- | --- | --- |
| Gouging Electrodes | SWISSO 15mm dia x 300mm graphite rod*  (Mumbai local manufactures, Make unknown)** | AVALLOY  ~10mm dia x 300 mm length | Carbon rods, 5mm diameter, (local welders, manufacturer unknown) | McMaster-Carr^(a)^  ~10mm dia x 300 mm length |
| 5-Volt SMPS | MY TRIDEV*  (DHRUV-PRO Led  Indicator 5 Volt / 40 Amp DC Output Power, AC Input 100/240vAC 200 Watts PSU)** | UNIT ELECTRONICS | KYSAN DC power supply HY3005D | From HP Desktop. Part number 366307-001 |
| Salt | Tata Iodized Table Salt*  (Tata Chemicals Ltd)** | Granulated Salt “La Fina” | Non-iodized Lab Salt | From a local market. non-iodized |
| Vinegar | Tops white synthetic vinegar*  (Kohena Agro White Vinegar)** | White Distilled Vinegar  “Clemente Jacques” | N/A^(b)^ | Kasaf Uganda, White Vinegar |
| Free Chlorine Measuring Equipment | N/A*^(c)^  (Hanna HI 38018)** | Hanna HI83300 multiparameter photometer | Titrimetric Method | Palintest. Contour Comparator |
| 1. ^Gouging rods sourced from the U.S.^ 2. ^Hydrochloric acid (HCl) was used for pH adjustments.^ 3. ^Unable to acquire due to access limitations.^   ^* Information corresponds to co-author Vijay Matange.^  ^** Information corresponds to co-authors at IIT Bombay.^ | | | | |
